# Effects of elevation of ANP and its deficiency on cardiorenal function

**DOI:** 10.1172/jci.insight.148682

**Published:** 2022-05-09

**Authors:** Daria V. Ilatovskaya, Vladislav Levchenko, Kristen Winsor, Gregory R. Blass, Denisha R. Spires, Elizaveta Sarsenova, Iuliia Polina, Adrian Zietara, Mark Paterson, Alison J. Kriegel, Alexander Staruschenko

**Affiliations:** 1 Department of Physiology, Medical College of Wisconsin, Milwaukee, Wisconsin, USA.; 2 Department of Physiology, Medical College of Georgia, Augusta University, Augusta, Georgia, USA.; 3 Department of Medicine, Division of Nephrology, Medical University of South Carolina, Charleston, South Carolina, USA.; 4 Department of Molecular Pharmacology and Physiology and; 5 Hypertension and Kidney Research Center, University of South Florida, College of Medicine, Tampa, Florida, USA.; 6 James A. Haley Veterans’ Hospital, Tampa, Florida, USA.

**Keywords:** Nephrology, Hypertension, Sodium channels

## Abstract

Atrial natriuretic peptide (ANP), encoded by *Nppa*, is a vasodilatory hormone that promotes salt excretion. Genome-wide association studies identified *Nppa* as a causative factor of blood pressure development, and in humans, ANP levels were suggested as an indicator of salt sensitivity. This study aimed to provide insights into the effects of ANP on cardiorenal function in salt-sensitive hypertension. To address this question, hypertension was induced in SS^NPPA–/–^ (KO of *Nppa* in the Dahl salt-sensitive [SS] rat background) or SS^WT^ (WT Dahl SS) rats by a high-salt (HS) diet challenge (4% NaCl for 21 days). Chronic infusion of ANP in SS^WT^ rats attenuated the increase in blood pressure and cardiorenal damage. Overall, the SS^NPPA–/–^ strain demonstrated higher blood pressure and intensified cardiac fibrosis (with no changes in ejection fraction) compared with SS^WT^ rats. Furthermore, SS^NPPA–/–^ rats exhibited kidney hypertrophy and higher glomerular injury scores, reduced diuresis, and lower sodium and chloride excretion than SS^WT^ when fed a HS diet. Additionally, the activity of epithelial Na^+^ channel (ENaC) was found to be increased in the collecting ducts of the SS^NPPA–/–^ rats. Taken together, these data show promise for the therapeutic benefits of ANP and ANP-increasing drugs for treating salt-sensitive hypertension.

## Graphical abstract

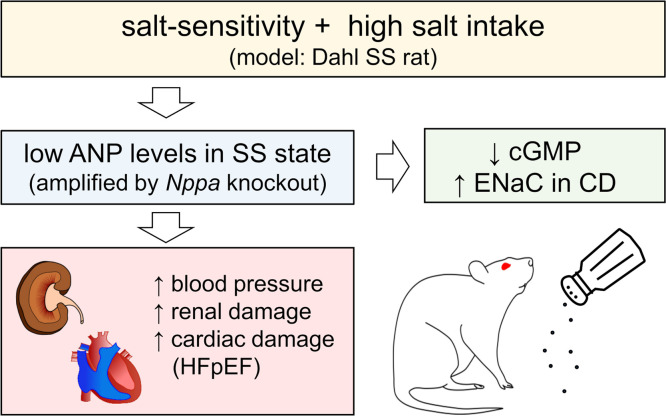

## Introduction

Hypertension is a significant public health concern; the Centers for Disease Control and Prevention estimates that 1 in every 3 Americans suffers from high blood pressure (BP) (1). While high dietary salt intake is a common risk factor for all types of hypertension, a subset of patients exhibits BP changes dependent on salt intake (2). Salt sensitivity is associated with a higher risk of pathological cardiometabolic events and organ damage. The mechanisms of salt-sensitive (SS) hypertension are not fully clear, and there are no specific treatments available for the SS subpopulation (3). Existing pharmacology is insufficient to properly control BP in SS patients (4), and there is a need to develop more effective therapies.

Atrial natriuretic peptide (ANP) has been proposed to play a role in the susceptibility to SS hypertension. Elevated ANP levels have been considered a hallmark for heart failure (HF) and nephrotic syndrome (5). ANP, encoded by the *Nppa* gene, is a hormone known to promote salt excretion, vasodilation, and BP reduction (6), in part through its antagonism of the renin-angiotensin-aldosterone system (RAAS) (7). *Nppa*^–/–^ mice displayed an SS phenotype (8). Furthermore, aldosterone-induced glomerular injury and proteinuria were more severe in *Nppa*^–/–^ mice (9). In rats, *Nppa* is located on the *Agtrap-Plod1* locus on chromosome 5, which has been associated with BP alterations and renal disease in several studies (10–12). In humans, ANP levels were suggested as a clinical marker of salt sensitivity (13, 14), and some studies show that SS hypertensive patients manifest a counterintuitive decrease in plasma ANP (15, 16).

In clinics, acute ANP infusion may improve cardiac function in HF patients (17). In 2015, the FDA approved a HF treatment drug featuring a combination of valsartan (an angiotensin receptor blocker [ARB]), and sacubitril (a neprilysin inhibitor, NEPi) (18). Sacubitril increases the circulating level of ANP by inhibiting the enzyme responsible for its degradation (neprilysin).

While the pathophysiology of SS hypertension is not completely clear, sodium imbalance resulting from renal pathology is crucial for developing this disease (19). The epithelial Na^+^ channels (ENaC) in the distal nephron are the limiting step of sodium reabsorption, and it was shown that inhibiting ENaC can attenuate salt sensitivity in animal models (20–22). There is a variety of endogenous stimuli that can regulate ENaC, including ANP (23–25). Importantly, the SS phenotype in *Nppa*^–/–^ mice was rescued by an ENaC inhibitor amiloride (26).

We hypothesized that deficient ANP signaling plays a role in the development of electrolyte imbalance and cardiorenal damage in SS hypertension. The study was designed 2-fold; first, we tested if ANP supplementation can alleviate renal and cardiac lesions in WT Dahl SS rats (SS^WT^) fed a high-salt diet (HS diet) to induce hypertension. Secondly, we tested the effects of the lack of ANP in an SS context using the *Nppa* KO generated in a Dahl SS rat background (SS^NPPA–/–^). This was achieved by precise analyses of the ANP-dependent BP changes, renal tubulointerstitial fibrosis, and natriuresis and diuresis, as well as by probing ENaC activity in the collecting ducts. These studies provide mechanistic insights into the effects of ANP on electrolyte homeostasis and cardiorenal function in the condition of SS hypertension.

## Results

### Continuous infusion of ANP ameliorates hypertension, renal fibrosis, and protein cast formation in the SS^WT^ rats.

**Figure 1:**
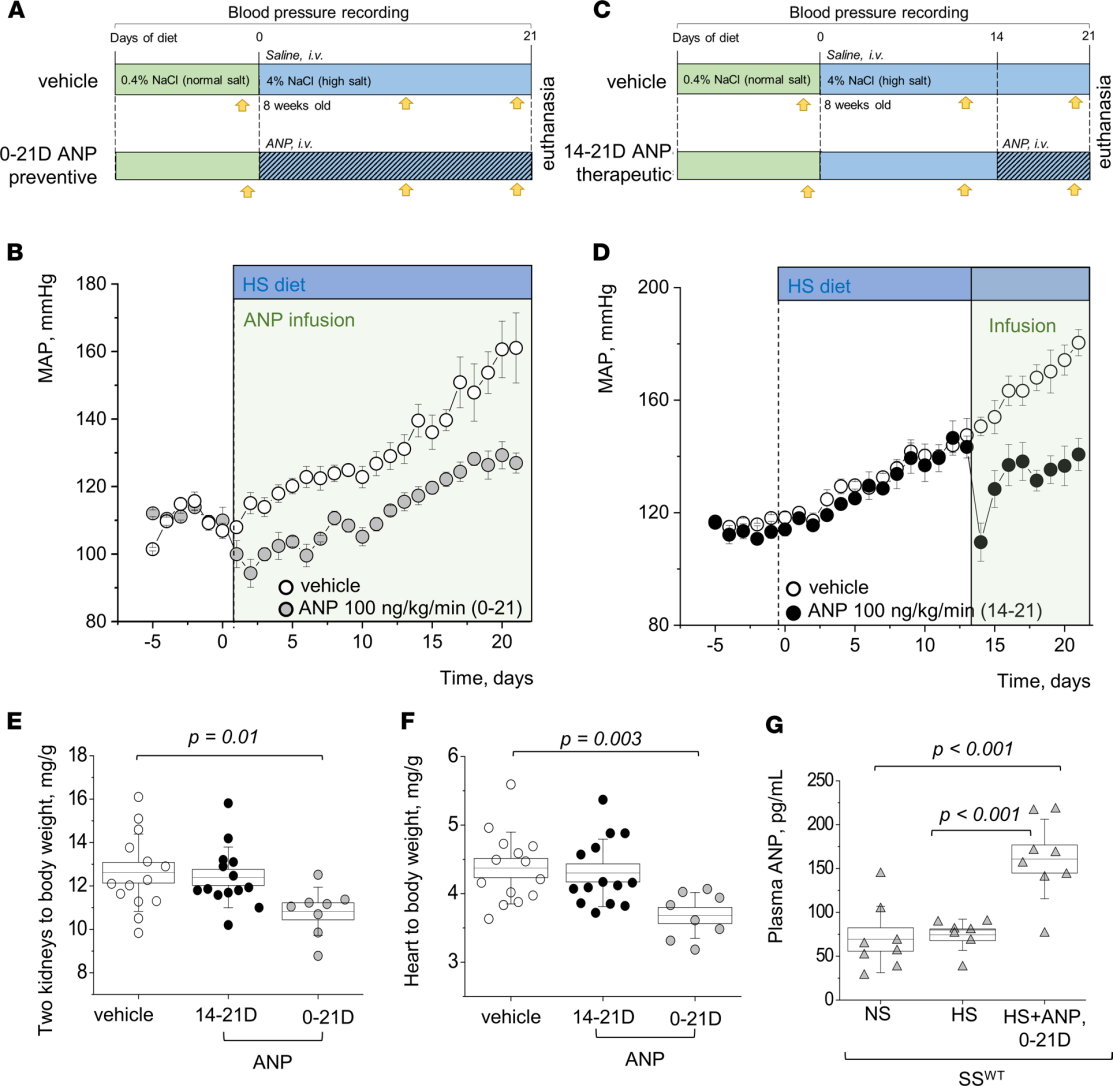
Both the therapeutic and preventive chronic infusion of ANP lower mean arterial pressure in male SS^WT^ rats. (**A**) The experimental protocol for the “preventive” infusion of ANP to the SS^WT^ rats; yellow arrows denote metabolic cage urine collections. (**B**) The mean arterial pressure (MAP) following i.v. infusion of 100 ng/kg/day ANP (*n* = 8) or vehicle (saline, *n* = 9) performed together with the 21-day HS diet challenge; *P* < 0.01 in HS diet–fed groups (vehicle versus infusion) starting day 1 of the infusion. (**C**) The experimental protocol for the “therapeutic” infusion of ANP to the SS^WT^ rats; yellow arrows denote metabolic cage urine collections. (**D**) I.v. infusion of 100 ng/kg/day ANP (*n* = 7) or vehicle (saline, *n* = 5) was started on day 14 of the 21-day HS diet challenge. *P* < 0.01 in HS diet–fed groups (vehicle versus infusion) starting day 1 of the infusion. (**E** and **F**) Graphs summarize 2 kidneys/body weight and heart/body weight ratios obtained at the end of the protocols. *n* = 14, 8, and 7 individual rats. Groups are the following: vehicle, rats infused with ANP for the last 7 days of the HS challenge (14–21 days), and throughout the HS challenge (0–21 days). (**G**) Plasma levels of ANP in the SS^WT^ rats fed a NS and a HS diet, and administered with 100 ng/kg/day ANP for 21 days during a HS challenge (HS and ANP, 0–21 days). Data were analyzed with 1-way ANOVA followed by a Holm-Sidak post hoc test; significant *P* values are shown on the graphs. Male animals were used.

**Figure 2:**
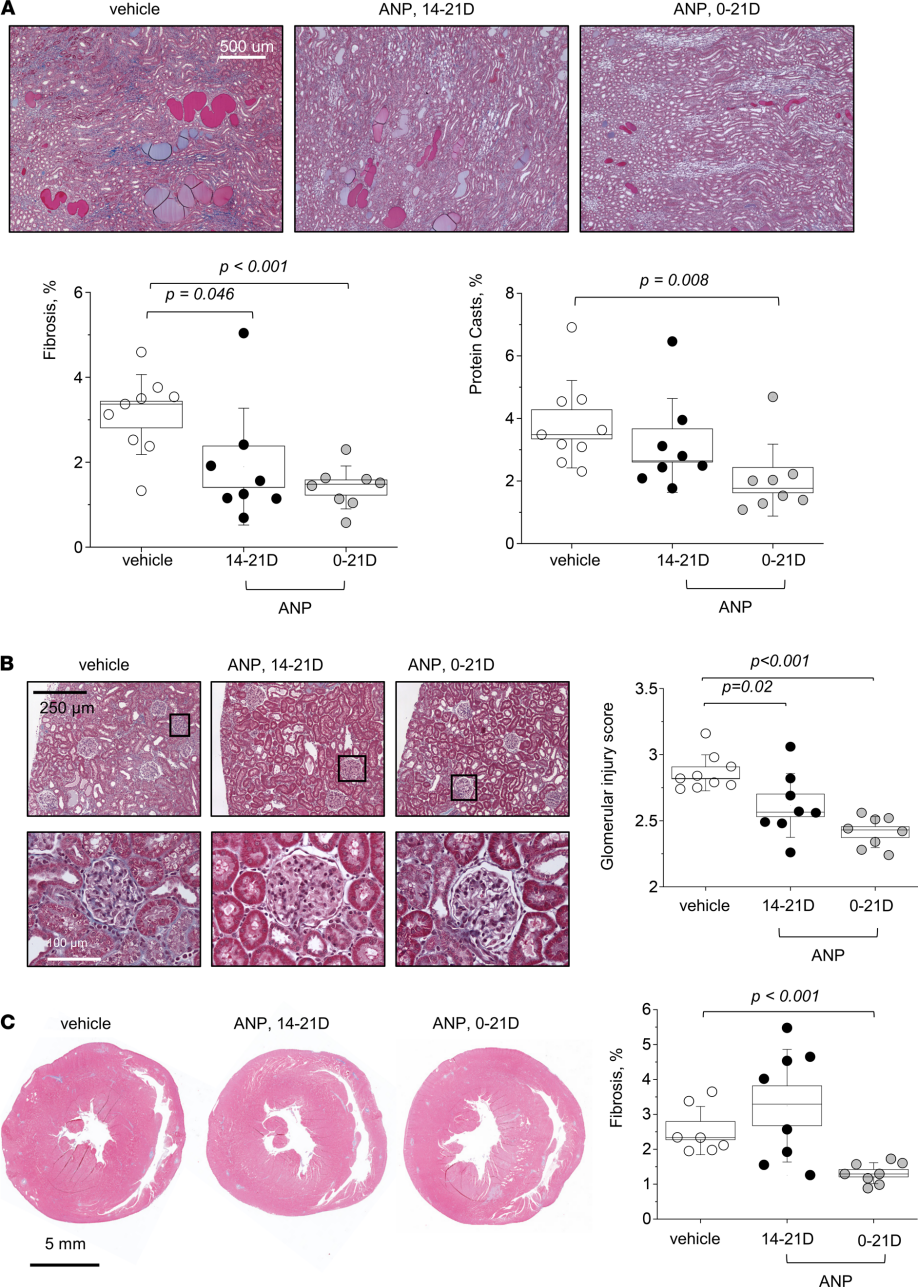
The therapeutic and preventive chronic infusion of ANP alleviate renal fibrosis and protein cast formation, as well as cardiac fibrosis, in male SS^WT^ rats. (**A**) Representative images from Masson trichrome–stained renal tissues of the SS^WT^ rats administered vehicle or ANP on days 14–21 and 0–21 of the HS challenge; shown are renal medullary images taken at 10×. Graphs summarizing renal fibrosis and protein casts formation (percentage of the total area of the kidney section) are shown below. *n* = 9, 8, and 8 independent tissues analyzed for the SS^WT^ rats administered vehicle or ANP on days 14–21 and 0–21. (**B**) Representative images from the cortical regions of the Masson trichrome–stained renal tissues of the SS^WT^ rats administered vehicle or ANP on days 14–21 and 0–21 of the HS challenge; shown are renal tissue images taken at 10×. Blindly scored glomerular injury is shown on the right; each point on the graph is an average of 100 randomly scored glomeruli from 1 rat. *n* = 9, 8, and 8 independent tissues analyzed for the SS^WT^ rats administered vehicle and ANP on days 14–21 and 0–21. (**C**) Representative images of Masson trichrome–stained cardiac tissues. The graph on the right summarizes cardiac interstitial fibrosis assessed in the SS^WT^ rats administered vehicle and ANP on days 14–21 and 0–21; *n* = 7, 8, and 8. Data were analyzed with 1-way ANOVA followed by a Holm-Sidak post hoc test; if found significant, *P* values are shown on the graphs. Male animals were used. Scale bars: 500 µm (**A**), 250 µm (**B**), and 5 mm (**C**).

According to the protocols shown in Figure 1, A and C, we continuously infused ANP i.v. into male SS^WT^ rats together with the administration of a HS (4% NaCl) diet for 21 days (Figure 1A) or starting on day 14 of the HS diet (Figure 1C). ANP infusion resulted in a protective effect in attenuating the mean arterial pressure (MAP) increase compared with the vehicle-infused group (Figure 1B). Furthermore, ANP infusion had a therapeutic effect, since it lowered MAP even when it was given when rats had already started to develop salt-induced hypertension (Figure 1D). ANP attenuated the increase in the kidney/body weight and heart/body weight ratios when ANP was administered during the last 7 days of the HS diet (Figure 1, E and F) and alleviated the organs’ hypertrophy further when ANP was delivered throughout the whole 21-day HS diet challenge (please note that, since the organs were not dehydrated, total body water could alter conclusions drawn from organ weights). Plasma ANP levels were significantly elevated in the infused group, confirming successful delivery of ANP (Figure 1G). Histological analysis (Figure 2) showed alleviation of the fibrosis, protein cast formation, and glomerular injury score (Figure 2, A and B) by ANP delivery during days 14–21 of the HS diet, and these parameters were further improved when ANP was infused for 21 days. Interestingly, cardiac fibrosis and heart hypertrophy were only alleviated when ANP was infused for the whole duration of the dietary challenge (Figure 1F and Figure 2C). Urine flow, electrolyte excretion, and plasma electrolyte levels were mostly similar among the groups, except for an increase in Na^+^ and Cl^–^ excretion on day 21 in the group of rats infused with ANP during days 0–21 (Supplemental Table 1; supplemental material available online with this article; https://doi.org/10.1172/jci.insight.148682DS1).

### SS^NPPA–/–^ rats exhibit higher BP, lower urine output and less sodium excretion compared with SS^WT^.

**Figure 3:**
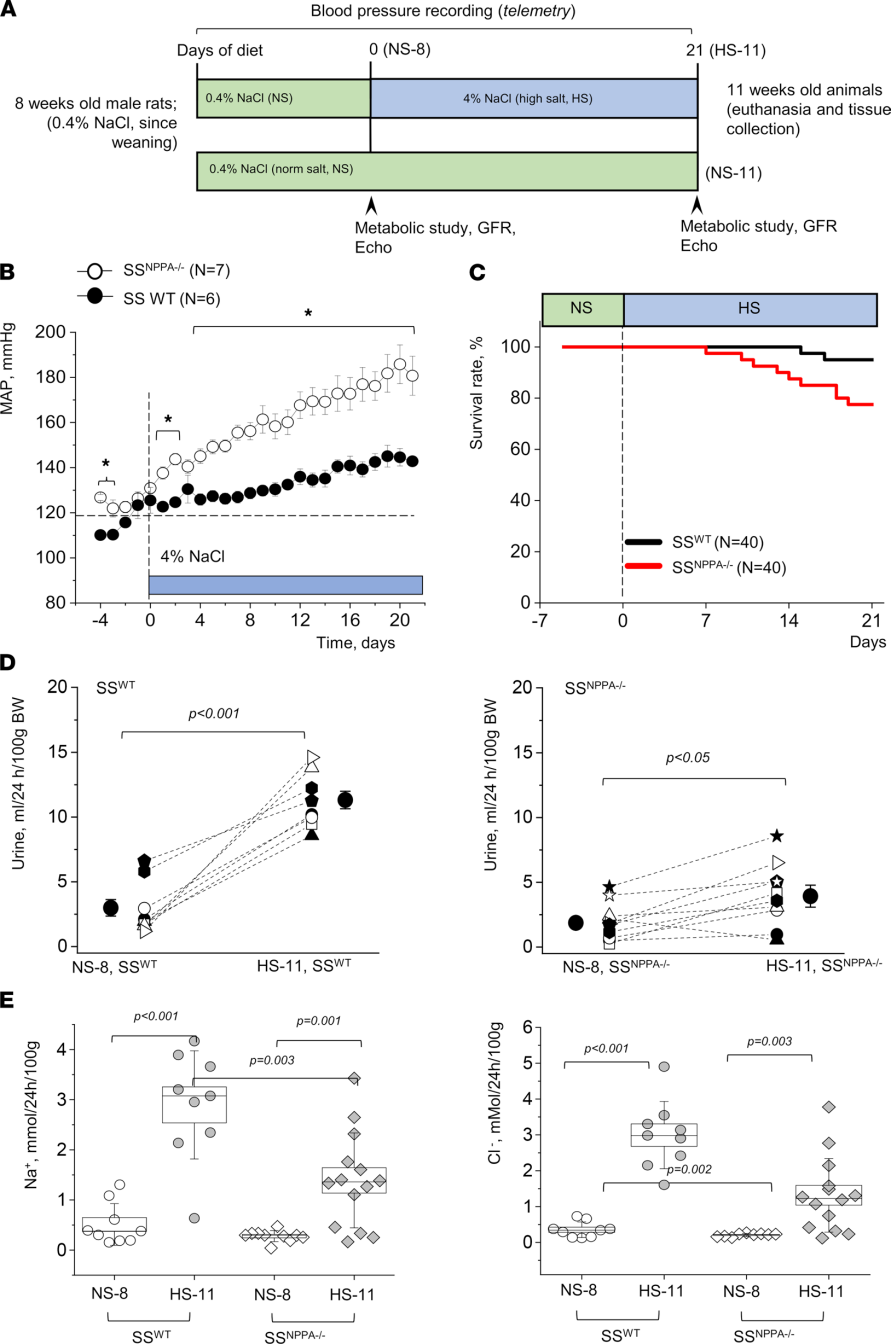
Male SS^NPPA–/–^ rats fed a HS diet exhibit higher blood pressure, reduced urine flow, and lower Na^+^ and Cl^–^ excretion than SS^WT^. (**A**) Schematic representation of the experimental protocol. (**B**) Mean arterial pressure (MAP) recorded via telemetry in SS^WT^ (*n* = 6) versus SS^NPPA–/–^ (*n* = 7) rats throughout the experimental protocol. *P < 0.05. (**C**) Survival rates of SS^WT^ versus SS^NPPA–/–^ rats during a HS diet challenge. (**D**) Urine flow in the 8- and 11-week-old animals (before and after starting the HS challenge). *n* = 9 for SS^WT^, *n* = 10 for the SS^NPPA–/–^ group. (**E**) Electrolyte excretion (Na^+^ and Cl^–^) in the 11-week-old animals (after NS since weaning, or NS until 8 weeks of age, and 3 weeks on HS, as shown in **A**). *n* = 9 for SS^WT^, *n* = 14 for the SS^NPPA–/–^ group. *P* values are shown on the graphs. NS-8 and NS-11 refer to 8- or 11-week-old animals fed NS since weaning, and HS-11 refers to 11-week-old animals fed a HS diet from week 8 until week 11. Data were analyzed with 2-way ANOVA (**E**), paired *t* test (**D**), log-rank test (**C**), or repeated-measures ANOVA (**B**), as appropriate. Male animals were used.

First, we assessed if lack of ANP changed the baseline BP or the development of hypertension compared with SS^WT^ animals. The experimental protocol is shown in Figure 3A. Before the initiation of the HS challenge, SS^NPPA–/–^ rats already exhibited elevated MAP in comparison with controls (123.4 ± 3.0 versus 110.3 ± 6.0 mmHg; *P* < 0.05; Figure 3B). During the HS challenge, the SS^NPPA–/–^ rats demonstrated a significantly higher MAP compared with SS^WT^; after 21 days on a HS diet, MAP was 185.8 ± 9.0 and 144.6 ± 4.0 mmHg for SS^NPPA–/–^ rats and SS^WT^ rats, respectively. Systolic and diastolic BP were both higher in the SS^NPPA–/–^ rats, while the circadian rhythms were largely undisturbed (Supplemental Figure 1, A and B). To examine if *Nppa* deficiency affects the circadian rhythm of BP, cosinor analysis of BP on the last 3 days of the normal salt (NS) and HS diets was performed. SS^NPPA–/–^ rats exhibited increased mesor on NS and HS, as expected due to their elevated MAP on both diets, while acrophase was similar between SS^WT^ and SS^NPPA–/–^ rats or between NS and HS. Amplitude was significantly increased in SS^NPPA–/–^ rats on HS, likely due to the already exacerbated MAP in this group.

When survival rates of the SS^WT^ versus SS^NPPA–/–^ rats were considered (Figure 3C), we observed an 18% lower survival of the KO animals on HS. It can be speculated that overall end-organ damage, HF (see below), or stroke contributed to a significant decline in survival. Interestingly, although we observed a small but statistically significant increase in plasma BNP level in the SS^NPPA–/–^ rats versus SS^WT^ rats on NS diet (Supplemental Figure 1C), this compensatory change was not present in the HS diet–fed animals. Therefore, it can be surmised that compensatory BNP increase was not a significant contributory factor to the changes observed during a HS challenge.

To test the involvement of ANP in fluid volume control, we compared diuresis and urinary electrolyte levels of 8-week-old SS^WT^ and SS^NPPA–/–^ rats on a NS diet, and again 21 days after the switch to a HS diet (Figure 3, D and E). The SS^NPPA–/–^ rats exhibited a lower 24-hour urine flow compared with SS^WT^ following the HS challenge. The SS^WT^ rats and SS^NPPA–/–^ on HS diet had an average 24-hour urine output of 11.32 ± 0.67 and 4.69 ± 0.85 mL, respectively (Figure 3D). Additionally, SS^NPPA–/–^ rats after the HS diet challenge exhibited decreased sodium and chloride excretion (Figure 3E). We report higher Ca^2+^ excretion on a HS diet (perhaps attributable to higher urine flow), but no differences were recorded between the genotypes (Supplemental Table 2). Creatinine excretion is reported in Supplemental Table 2. Body weights were similar between SS^WT^ and SS^NPPA–/–^ groups when fed the same diet (Supplemental Table 2). Furthermore, water consumption was assessed, and similar values were observed between the genotypes, while HS diet induced a significant increase in the amount of water the animals drink, as expected (for age-matched SS^WT^ and SS^NPPA–/–^ rats on NS, respectively: 19.4 ± 1.4 and 20.1 ± 1.4 mL/day, and for age-matched SS^WT^ and SS^NPPA–/–^ rats on HS, respectively: 37.9 ± 1.4 and 35.6 ± 2.2 mL/day; *P* value for dietary challenge < 0.001 for both genotypes).

### Plasma electrolytes and metabolic parameters.

Plasma levels of alanine aminotransferase (ALT) and alkaline phosphatase (Alk.Ptase) (Supplemental Table 2) were decreased by a HS diet in the SS^WT^ rats, while in the SS^NPPA–/–^ rats, these values were higher at baseline and not changed by the HS diet. In addition, we report an increase in cholesterol in the SS^WT^ animals on a HS diet, not observed in the SS^NPPA–/–^ rats (Supplemental Table 2). Plasma Na^+^ level was significantly increased in the SS^NPPA–/–^ rats on both NS and HS diets, and in SS^WT^ rats on a HS diet, in comparison with SS^WT^ rats fed NS diet, indicating retention that accompanies BP increase in SS^WT^ rats on a HS diet and SS^NPPA–/–^ rats on either diet (Supplemental Table 3). Cl^–^ and K^+^ plasma levels were similar among all groups. At 11 weeks of age, plasma creatinine was elevated in SS^NPPA–/–^ rats fed a NS diet, while blood urine nitrogen (BUN) was similar across the groups diet (Supplemental Table 3). Hematocrit was tested in 8-week-old animals at baseline, on NS as an indicator of intravascular fluid volume; interestingly, SS^NPPA–/–^ rats had hematocrit of 43.2% ± 1.6% versus 46% ± 1.2% in SS^WT^ animals (*P* < 0.05).

### Glomerular lesions and proteinuria-related changes in SS^NPPA–/–^ versus SS^WT^ rats.

**Figure 4:**
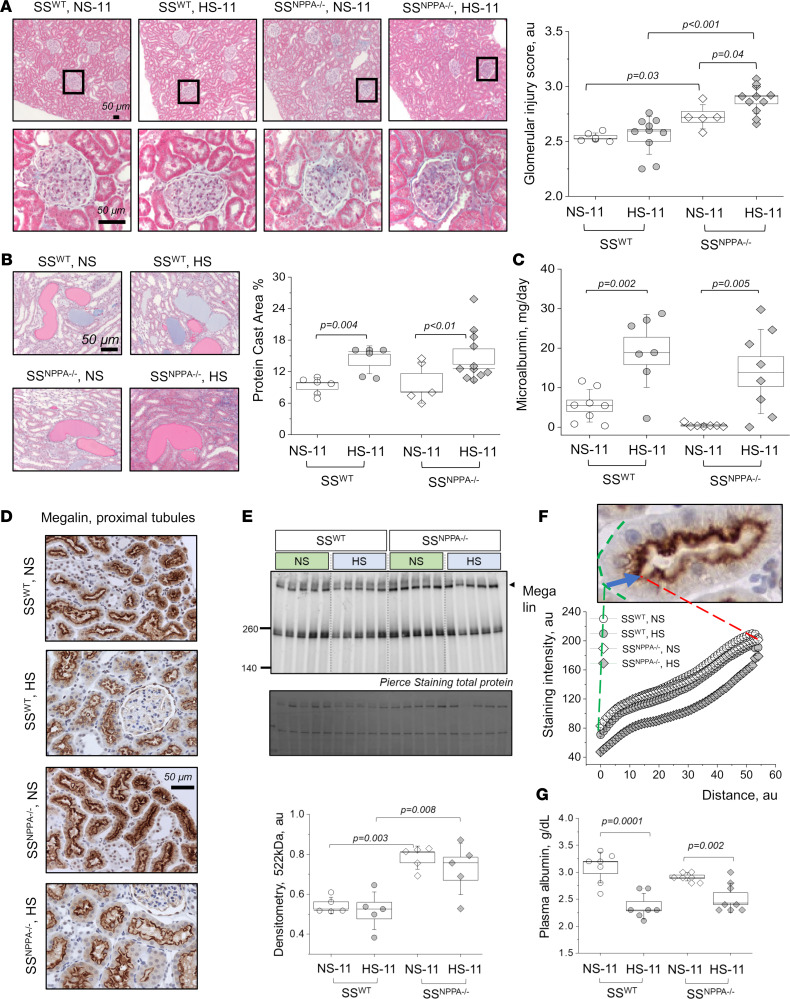
ANP deficiency in male rats exacerbates glomerular damage but does not aggravate microalbuminuria and renal protein cast formation induced by a HS diet. (**A**) Representative cortical images from Masson trichrome–stained kidneys taken at 10× (upper row), and expended regions of interest (bottom row). Blind scores are shown on the right; each point on the graph is an average of 100 scored glomeruli from 1 rat. *n* = 6, 10, 5, and 11 independent tissues scored for the SS^WT^ rats on NS and HS, and SS^NPPA–/–^ rats on NS and HS. (**B**) Representative images from Masson trichrome–stained renal tissues showing protein casts formation. Shown are images taken at 20×; graph on the right summarizes the percentage of the protein cast area to whole kidney section area; *n* = 6, 6, 5, and 11 independent tissue scans analyzed for the SS^WT^ rats on NS and HS, and SS^NPPA–/–^ rats on NS and HS. (**C**) Urinary microalbumin excretion (normalized to urine flow). *n* = 8, 7, 7, and 8 urine samples from independent animals were analyzed for the SS^WT^ rats on NS and HS, and SS^NPPA–/–^ rats on NS and HS. (**D**) Representative staining for megalin in the renal cortex (20×). (**E**) Western blot analysis of megalin expression in the renal cortex of the SS^WT^ rats on NS and HS, and SS^NPPA–/–^ rats on NS and HS; each lane represents an independent renal tissue sample obtained from a different rat. *n* = 5 per group. (**F**) Intensity profiles of megalin staining assessed in the cortical proximal tubules of the SS^WT^ rats and SS^NPPA–/–^ rats on NS and HS. *n* = 3 animals per group; at least 8 random images at 40× were analyzed per animal, with *n* = 8–10 individual tubules measured per image (total *n* = 172, 238, 217, 229 in SS^WT^ rats and SS^NPPA–/–^ rats on NS and HS). (**G**) Plasma albumin levels obtained from SS^WT^ rats on NS and HS, and SS^NPPA–/–^ rats on NS and HS; *n* = 7, 7, 8, 8. Data were analyzed with 2-way ANOVA or repeated measures ANOVA; if significant, *P* values are shown on the graphs. Male animals were used. Scale bars: 50 μm.

Injury scoring revealed exacerbated glomerular damage in the SS^NPPA–/–^ rats on either diet versus SS^WT^ animals (Figure 4A). Despite the higher glomerular injury score, protein casts formation and microalbuminuria were not different in SS^NPPA–/–^ rats compared with SS^WT^, although the HS diet augmented these parameters in both genotypes (Figure 4, B and C). In line with the findings for the microalbuminuria and protein casts, when fed a HS diet, both SS^WT^ and SS^NPPA–/–^ rats exhibit decreases in plasma albumin (Figure 4G) and plasma total protein levels, as well as changes in albumin-globulin ratio and plasma globulin (Supplemental Table 2). To further characterize these findings, we assessed megalin expression in the renal cortex using IHC (Figure 4D) and Western blot (Figure 4E). Furthermore, we assessed the intensity profiles for megalin along a line drawn from the basolateral membrane to the apical (Figure 4F). Western blotting revealed overall higher megalin levels in SS^NPPA–/–^ rats. When changes in the slopes of the intensity plots in Figure 4F were quantified along the brush border (reflecting the apical distribution of megalin), we observed that the slope at the brush border is much steeper in the SS^NPPA–/–^ rats fed a HS diet, compared with the other groups, which is consistent with the example images in Figure 4D. The slope of the curve quantifying megalin distribution at the brush border, in the SS^WT^ and SS^NPPA–/–^ rats on NS, respectively, was 1.32 ± 0.23 and 1.89 ± 0.11. The slopes in the SS^WT^ and SS^NPPA–/–^ rats on HS, respectively, were 1.42 ± 0.27 and 3.95 ± 0.2 (*P* value for dietary challenge < 0.05 when SS^NPPA–/–^ rats on either diet were compared with SS^WT^). These data indicate that, in SS^NPPA–/–^ rats, sorting of megalin is more apical than in SS^WT^ rats. As expected, GFR declined in the SS^WT^ rats after a HS challenge; however, no pronounced decrease was found in the SS^NPPA–/–^ group (Supplemental Figure 2).

### Cardiac function in the SS^NPPA–/–^ rats with the HFpEF phenotype.

**Figure 5:**
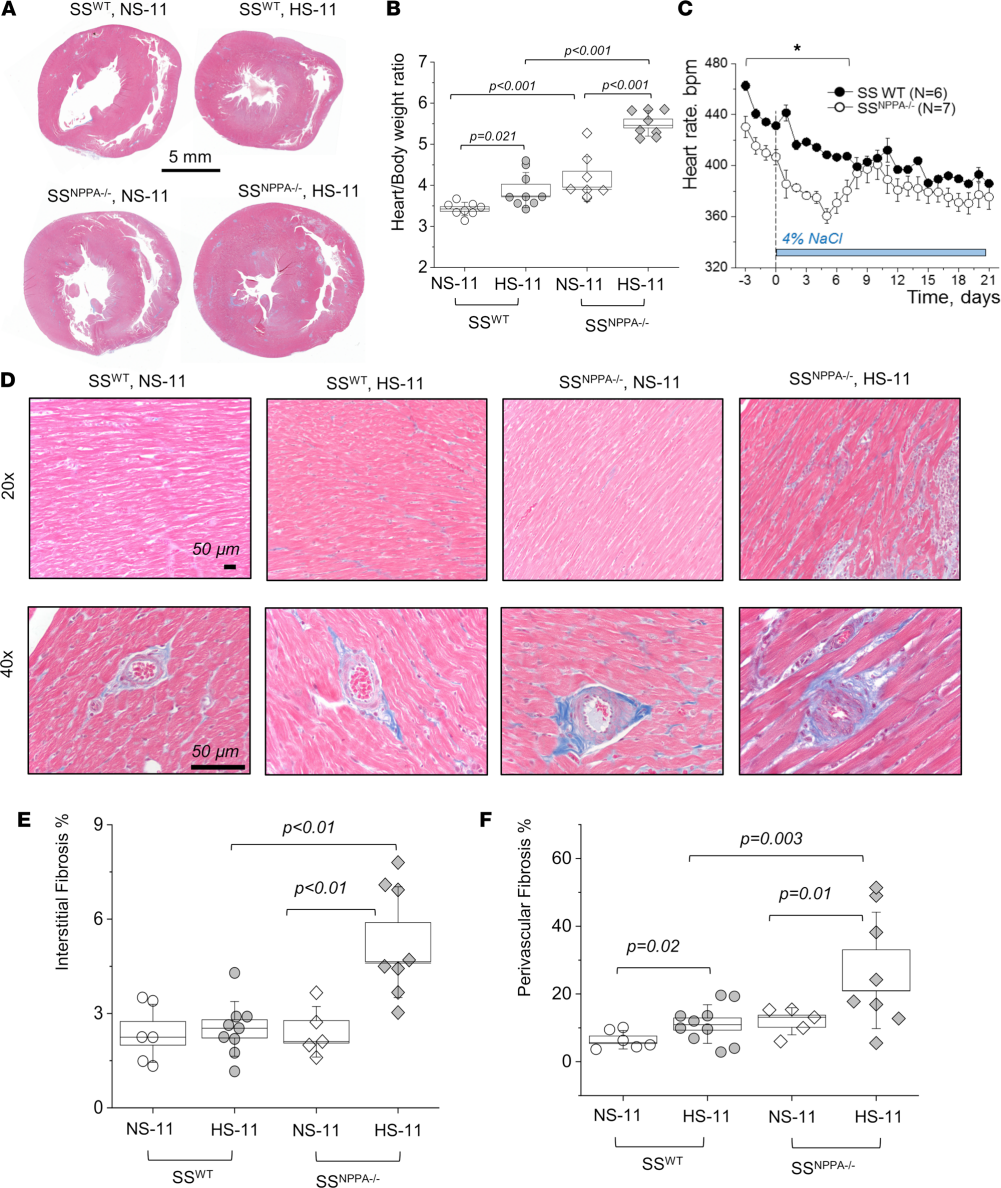
Male SS^NPPA–/–^ rats exhibit aggravated cardiac fibrosis and hypertrophy following a HS diet challenge. (**A**) Masson trichrome–stained cardiac tissues. (**B**) Summary of heart/body weight ratio obtained from the 11-week-old SS^WT^ rats on NS and HS, and SS^NPPA–/–^ rats on NS and HS; *n* = 8, 9, 8, 8. (**C**) Continuous heart rate recorded with telemetry from the SS^WT^ (*n* = 6) and SS^NPPA–/–^ (*n* = 7) rats throughout the protocol (each point shows daily averages from 9 a.m. to 12 p.m.). (**D** and **E**) Representative images of Masson trichrome–stained hearts and analysis of cardiac interstitial fibrosis assessed in the 11-week-old SS^WT^ rats on NS and HS, and SS^NPPA–/–^ rats on NS and HS; *n* = 6, 9, 5, and 8 rats (independent tissues analyzed). Fibrosis was calculated as percentage of the total area of the tissue section. (**F**) Perivascular cardiac fibrosis assessed in the 11-week-old SS^WT^ rats on NS and HS, and SS^NPPA–/–^ rats on NS and HS, presented as percentage of fibrotic area versus whole vessel area; *n* = 6, 9, 5, 8 (each point represents an average of 25–30 randomly selected vessels per tissue section). Data were analyzed with 2-way ANOVA, followed by a Holm-Sidak post hoc test; if significant, *P* values are shown on the graphs. Male animals were used.

**Figure 6:**
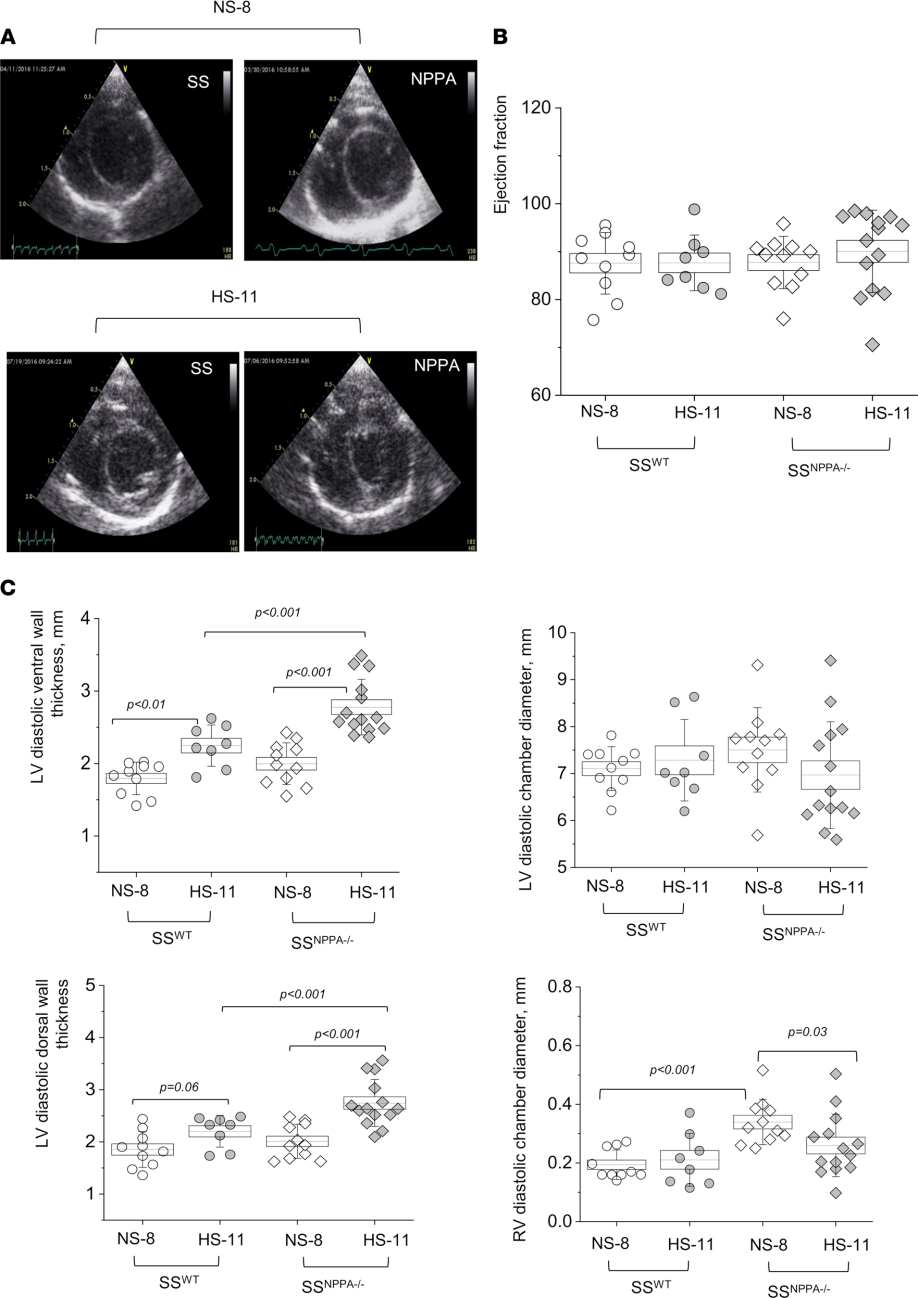
Echocardiography demonstrates preserved ejection fraction and fractional shortening in the SS^NPPA–/–^ rats. (**A**) Representative short-axis images of the SS^WT^ and SS^NPPA–/–^ rat hearts (8 weeks old on NS before, and 11 weeks old after the HS diet challenge) in end of diastole obtained by echocardiography. (**B** and **C**) Summary graphs show ejection fraction (**B**) and diastolic wall thickness and chamber diameters (**C**) obtained from these groups. *n* = 6, 9, 5, 8. Data were analyzed with repeated-measures ANOVA; if significant, *P* values are shown on the graphs. LV, left ventricle; RV, right ventricle. Male animals were used.

**Figure 7:**
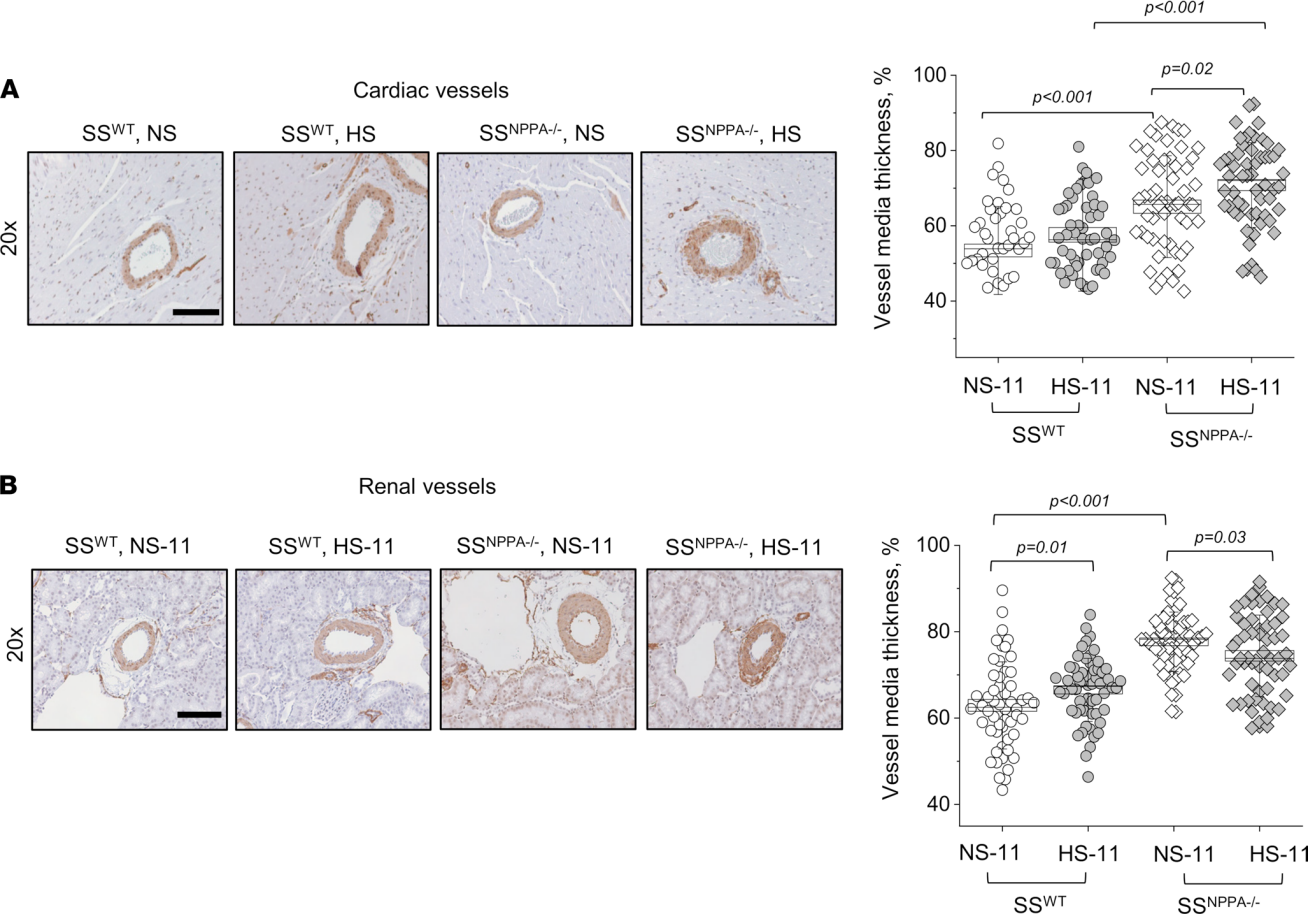
Male SS^NPPA–/–^ rats show more pronounced renal and cardiac vessel media thickness on both NS diet and after a HS diet challenge, compared with the SS^WT^ rats. (**A** and **B**) Representative images of cardiac (**A**) and renal (**B**) tissues stained for αSMA (brown), obtained at 20×. The graphs on the right summarize the vessel media thickness calculated from the αSMA staining in the 11-week-old SS^WT^ rats on NS and HS, and SS^NPPA–/–^ rats on NS and HS. *n* = 3 individual animals analyzed per group; *n* = 54, 60, 60, 60, for the cardiac vessels; and *n* = 56, 60, 60, 60 for renal vessels, in SS^WT^ rats on NS and HS, and SS^NPPA–/–^ rats on NS and HS (show are data points from individual vessels). Data were analyzed with 2-way ANOVA, followed by a Holm-Sidak post hoc test; if significant, *P* values are shown on the graphs. Male animals were used. Scale bars: 200 µm.

First, we assessed the hypertrophy of the cardiac tissues. As seen in representative scans shown in Figure 5A, and summarized in Figure 5B, there is a significant increase in heart size in the SS^WT^ rats fed a HS dies (versus NS), as well as in the SS^NPPA–/–^ rats at baseline versus SS^WT^. The most prominent hypertrophy was observed at the end of the HS diet challenge in the SS^NPPA–/–^ group, where the heart/body weight ratio reached 5.5 ± 0.1 mg/g (versus 3.8 ± 0.1 mg/g in the HS diet–fed SS^WT^ group; please note that, since the organs were not dehydrated, total body water could alter conclusions drawn from organ weights). Continuous heart rate (HR) measurement via telemetry demonstrated a significantly lower HR in the SS^NPPA–/–^ group at baseline compared with the SS^WT^ group (431 ± 6 versus 465 ± 4 bpm), which persisted until day 7 of the HS diet challenge, when this compensatory effect was lost (Figure 5C). Shown in Supplemental Figure 3 are the circadian rhythms of the HRs. Masson trichrome staining showed a remarkable increase in the percentage of interstitial and perivascular fibrosis in the SS^NPPA–/–^ group versus all other groups (Figure 5, D–F). Echocardiography analysis demonstrated that, most notably, there were no changes in the ejection fraction and fractional shortening, irrespective of the diet or genotype (Figure 6 and Supplemental Table 4). Other significant differences were detected in echocardiography; for instance, genotype-dependent increase in the left ventricle (LV) systolic and diastolic ventral wall thickness, LV systolic and diastolic dorsal wall thickness, and right ventricle (RV) systolic and diastolic chamber diameters in the SS^NPPA–/–^ rats versus SS^WT^ rats. Analysis of the cardiac α-smooth muscle actin (αSMA) staining revealed an increase in vessel media thickness in the SS^NPPA–/–^ group, independent of the diet (Figure 7A). Interestingly, the same vascular damage pattern was observed in the renal vessels (Figure 7B).

### RAAS profile and cGMP levels in the absence of ANP.

**Figure 8:**
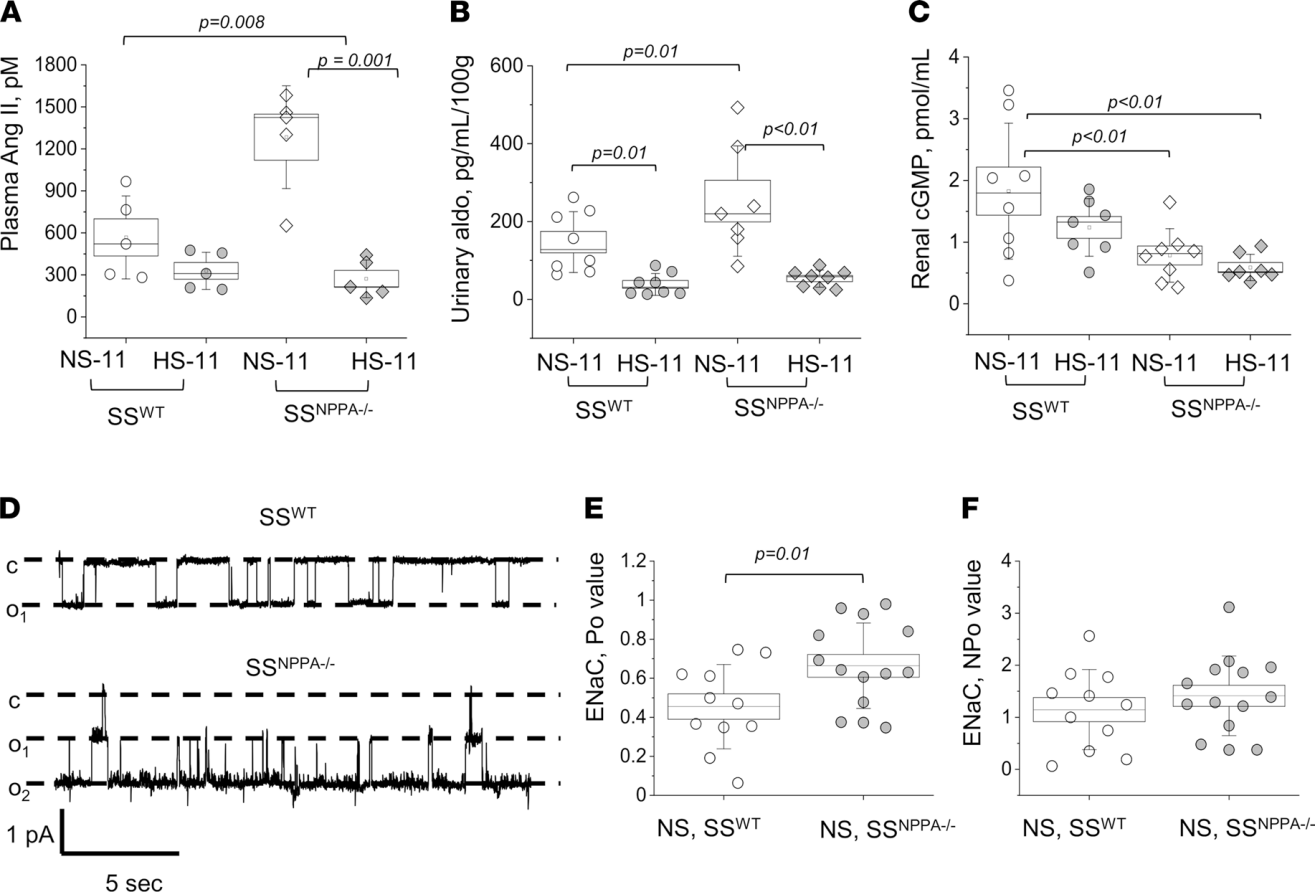
RAAS levels, cGMP concentration and ENaC activity in the SS^NPPA–/–^ rats. (**A**) Plasma level of Angiotensin II (Ang II) in the 11-week-old SS^WT^ rats on NS and HS, and SS^NPPA–/–^ rats on NS and HS. *n* = 5 samples obtained from individual rats, for all groups. (**B**) Urinary aldosterone excretion (normalized to daily urine flow) in the 11-week-old SS^WT^ rats on NS and HS, and SS^NPPA–/–^ rats on NS and HS. *n* = 8, 8, 7, 8 samples obtained from individual rats. (**C**) cGMP level in the renal tissues of the 11-week-old SS^WT^ rats on NS and HS, and SS^NPPA–/–^ rats on NS and HS. *n* = 8, 7, 8, 7 tissue samples obtained from individual rats. Data in **A**–**C** were analyzed with 2-way ANOVA followed by a Holm-Sidak post hoc test; if significant, *P* values are shown on the graphs. (**D**) Representative single-channel traces for ENaC activity recorded in freshly isolated split opened CCDs from 11-week-old SS^WT^ and SS^NPPA–/–^ rats. Patches were held at the test potential of V_h_= −V_p_= –40 mV. Inward Li^+^ currents are downward. The closed and open states of the channel are denoted by c and o_i_, respectively. (**E** and **F**) Summarized single-channel open probability (*P_o_*) and *NP_o_* of the recorded ENaC channels. In **E** and **F**, data was analyzed with a 2-tailed *t* test; if significant, *P* values are shown on the graphs. *n* = 7 individual rats per group; *n* = 11 and 14 recordings obtained in SS^WT^ and SS^NPPA–/–^ rats on NS, respectively. Male animals were used.

We tested the levels of aldosterone and angiotensin (Ang) metabolites in plasma of SS^WT^ versus SS^NPPA–/–^ rats fed a NS and HS diets. As summarized in Figure 8A, SS^WT^ rats displayed relatively low levels of plasma Ang II, which was reduced (not significantly) by a HS diet. A similar trend was observed for all other tested Ang metabolites (Ang I, Ang II, Ang 1–7, Ang 1–5, and Ang IV; Supplemental Table 3). As expected, at baseline (on NS diet), the *Nppa* KO resulted in a significant increase in all these RAAS components (Figure 8A and Supplemental Table 3), but RAAS metabolites were remarkably suppressed after the HS diet challenge. RAAS suppression in the SS^NPPA–/–^ rats on the HS diet was further confirmed by measuring urinary aldosterone, which exhibited a similar increase in the SS^NPPA–/–^ rats on NS (versus SS^WT^) and suppression on a HS diet (Figure 8B). RAAS and nitric oxide pathways are very closely interlinked, mutually regulated, and share many downstream effectors and cascades (27). Furthermore, ANP receptors are membrane-bound guanylyl cyclase receptors; thus, it was interesting to test if the lack of ANP leads to changes in cyclic guanosine monophosphate (cGMP) accumulation. As shown in Figure 8C, cGMP level is significantly lower in the SS^NPPA–/–^ rats, both on HS and NS diets.

### ENaC activity is affected by the KO of Nppa.

We applied single-channel patch-clamp electrophysiology in the isolated split opened cortical collecting ducts (CCD) from SS^WT^ and SS^NPPA–/–^ rats fed a NS diet and directly measured the activity of the ENaC in CCD. As shown in Figure 8, D–F, *Nppa* KO caused a significant increase in single-channel open probability of ENaC (*P_o_*); however, the *NP_o_* of the channel was not different between the SS^WT^ and SS^NPPA–/–^ groups. This implicates sodium reabsorption via ENaC channels in the sodium retention and BP increase in the SS^NPPA–/–^ rats. Observed changes in ENaC are likely mediated by the channels’ activity rather than the number of the channels in the plasma membrane of the CCD. Thus, we conclude that, in the distal nephron, lack of ANP results in higher sodium reabsorption via ENaC.

## Discussion

Our data demonstrate that the KO of *Nppa* exacerbates renal disease progression and hypertension development in Dahl SS rats, while a chronic i.v. infusion of ANP attenuated the hypertensive phenotype. These results suggest that supplementation of ANP may be a means to improve renal function and BP control in SS hypertension. It should be emphasized that, although chronic (21-day) ANP infusion in our study resulted in alleviation of BP and renal hypertensive lesions in the SS rats, direct translation of these findings into a clinical setting is controversial, although it was reported that, in response to HS intake, secretion of ANP may be blunted in African American/Black SS hypertensive individuals (8, 15, 28, 29). The Dallas Heart study showed that African American/Black individuals had lower natriuretic peptide levels than White and Non-Black Hispanic patients, concluding that this may lead to greater susceptibility to hypertension (30). Information derived from the Framingham Offspring Cohort was able to predict SS hypertension by lower levels of circulating N-terminal ANP (31). We have reported earlier that a valsartan/sacubitril combination may be beneficial for SS hypertension (32), as its administration to Dahl SS rats resulted in somewhat beneficial effects on renal tubulointerstitial fibrosis and protein cast formation; however, sacubitril and other NEPi can affect the level of many peptides other than ANP, including bradykinin, Ang II, and endothelin-1 (33–36). In 2015, a clinical trial tested the effects of sacubitril/valsartan compared with valsartan in Asian patients with SS hypertension (37). The authors reported that, over a 4-week period, sacubitril/valsartan combination resulted in more effective natriuresis and diuresis, better BP control, and significantly reduced N-terminal proBNP levels than valsartan alone. In summary, although there is some promise, great care should be exercised before using NEPi or ANP infusion in hypertensive patients, and more specific pharmacology should be developed to target ANP levels with higher precision.

We demonstrated that cGMP level is decreased in the SS^NPPA–/–^ rats fed a HS diet, versus control animals; we hypothesize that this decrease is an important contributor to the observed phenotype. The role of cGMP and ANP in salt sensitivity has been studied (38–41), and impairment in cGMP generation was reported in the Dahl SS rats (42, 43). Administration of ANP, its derivatives, or L-arginine were shown to attenuate SS hypertension (44). Thus, our results support the notion that the renal ANP/cGMP axis has a crucial role in SS hypertension.

Next, we report reduced sodium excretion in the SS^NPPA–/–^ rats compared with SS^WT^. Natriuretic and diuretic actions of ANP affect a variety of renal channels and transporters along the nephron. In CCD, ANP was shown to reduce sodium reabsorption by inhibiting transient receptor potential-vanilloid 4 channel (TRPV4) and polycystin 2, and by decreasing vasopressin-induced water reabsorption (23). ENaC in the CCD also can be regulated by ANP and/or cGMP (39); however, the mechanism for this regulation is not fully characterized (24). Guo et al. have shown in *Xenopus* 2F3 cells that ANP, via a cGMP-mediated pathway, can inhibit ENaC, which is consistent with the natriuretic effect of ANP. However, Yamada et al. (45, 46) demonstrated that ANP and cGMP activated ENaC-dependent sodium transport in frog epithelial cells. On the other hand, Poschet et al. (47) reported that intracellular cGMP inhibited ENaC activity in human bronchial cells; Zhao et al. (6) have shown that ANP increased sodium delivery in the distal nephron. In our study, in the SS hypertension context, ENaC *P_o_*, but not the number of channels, was increased in the SS^NPPA–/–^ versus SS^WT^ rats. Our data led us to hypothesize that decreased levels of ANP or a lack of ANP result in lower cGMP production, which stimulates ENaC activity in the CCD, thus increasing sodium reabsorption and leading to sodium retention.

In some studies, ANP was shown to antagonize the effects of RAAS (48), cooperate with the dopaminergic system (49), and affect vasopressin (23), endothelin-1 (50), and kallikrein (51) functions. To our knowledge, we provide the first direct evidence of changes in RAAS in the absence of ANP. At baseline, lack of ANP resulted in an increase in RAAS components; however, HS diet suppressed RAAS both in the SS^WT^ and in the SS^NPPA–/–^ animals. Interestingly, SS hypertension in *Nppa*-KO mice was prevented by AT1 receptor antagonists (52, 53); however, ARBs are considered inferior in efficacy for treatment of SS patients (54). Taking into consideration that RAAS components were significantly blunted in both SS^WT^ and the SS^NPPA–/–^ rats fed a HS diet, this might explain the low efficacy of ARBs in SS hypertension. Ang II is known to stimulate the activity of ENaC in the distal nephron (55, 56). Indeed, we observed increased ENaC activity in the SS^NPPA–/–^ rats versus SS^WT^ rats. However, long-term Ang II administration induces translocation of ENaC toward the apical membrane (55), and we did not record changes in ENaC number. 

In addition, our results should be considered in the context of the cardiac effects of ANP. Our data show that, while there is an elevation in cardiac interstitial and perivascular fibrosis in SS^NPPA–/–^ animals in comparison with SS controls, there was no evidence of changes in fractional shortening and ejection fraction. The data point toward a HF with preserved ejection fraction (HFpEF) phenotype in the SS^NPPA–/–^ rats. Interestingly, the majority of patients with a HFpEF diagnosis are hypertensive (57). As a new animal model of HFpEF, the SS^NPPA–/–^ rat presents a unique opportunity to study the complex cardiorenal interaction in hypertension (58). According to a recent metaanalysis that compared sacubitril/valsartan (ARNi) combination with traditional drugs, ARNi significantly improved echocardiography and HF biomarkers, and it reduced the incidence of hyperkalemia and renal dysfunction in patients with chronic HF (59). However, it is important to emphasize that caution should be exercised when prescribing NP-based drugs to HF patients, especially in the context of the available B-type natriuretic peptide–related (BNP-related) data. When BNP and its analogs, such as nesiritide, were administered to HF patients, they were reported to potentially worsen renal function and increase the risk of short-term mortality (60–62).

We would like to acknowledge the limitations of this study. Here, we used a KO of *Nppa*; however, it might not be entirely an ANP-specific loss-of-function approach, since ANP level changes are closely linked to other components of the NP system such as BNP (encoded by *Nppb*) and should be considered in the context of potential compensatory changes. *Nppb* KO in the Dahl SS rat background resulted in adult-onset hypertension, increased left ventricular mass with hypertrophy, and progressive nephropathy with proteinuria, fibrosis, and glomerular alterations (63). This manuscript confirmed the critical role of BNP in the development of systemic hypertension. As demonstrated in our experiments, there is a subtle compensatory increase in BNP level in the SS^NPPA–/–^ group versus WT rats on NS; however, values were similar between the groups fed a HS diet. Therefore, it can be surmised that the compensatory BNP increase is not a significant contributory factor to the changes observed during a HS challenge.

The putative defects leading to the development of SS hypertension involve a variety of pathways, including impaired mechanisms that assist in excreting a salt load (e.g., NPs, renal eicosanoids, endothelin system, nitric oxide bioavailability) or, conversely, lack of suppression of an antinatriuretic system (for instance, RAAS or abnormal activity of renal channels and transporters) or involvement of the SNS and epigenetics (2, 64). Oxidative stress and immune cell infiltration are among the widely discussed mechanisms important for SS hypertension (65, 66). ANP has been reported to lead to production of the reactive oxygen species (ROS) through intracellular signaling pathways involving DAG and cGMP (67, 68). It can be thus surmised, given the lower level of cGMP that we report in the SS^NPPA–/–^ rats, that a shift in ROS balance toward free radical production and a decrease in NO bioavailability may be an important contributing factor to the amplification of the hypertensive disease when ANP levels are low. Furthermore, evidence suggests that there is a mechanistic connection between NPs and mitochondrial bioenergetics (69), which — given the established role of mitochondria in SS hypertension and renal function — allows for the proposal of future studies of the ANP/mitochondria axis. Although there are limited data to support the direct involvement of ANP in the immune response, NPRA (receptor for ANP and BNP) has been shown to be important for the inflammatory processes during tumorigenesis (70). Some studies proposed ANP to be a cardiovascular cytokine rather than a hormone, since it is able to contribute to innate immunity by stimulating the host defense against extracellular microbes, inhibition of the synthesis and release of proinflammatory markers, and inhibition of the expression of adhesion molecules (71). ANP has also been reported to contribute to the adaptive immunity by reducing the number of CD4^+^CD8^+^ lymphocytes, increasing the CD4^–^CD8^–^ cells and stimulating the differentiation of naive CD4^+^ cells toward the Th2 and/or Th17 phenotypes (71). Although we have not explored the immune system–related ramifications of the *Nppa* KO in this study, it is a plausible future avenue of research in the context of SS hypertension, which is a disease accompanied with renal infiltration of immune cells (66, 72–74).

Overall, our data allow us to conclude that ANP infusion is beneficial for the alleviation of renal tubulointerstitial fibrosis, protein cast formation, and BP in the condition of salt sensitivity. Mechanistically, we report that, in SS hypertension, ANP deficiency as a result of a KO of *Nppa* led to lower production of cGMP. We can surmise from these findings that decreased cGMP levels failed to inhibit ENaC activity in the CCD, which in turn increased sodium reabsorption and resulted in more pronounced hypertension. These findings suggest that medications that increase ANP levels, or specific NPRA agonists, may improve renal function in SS individuals with hypertension. A recent manuscript by Chen et al. introduced a first-in-human study of MANP (an ANP analog) in human hypertension (75). The authors reported that MANP was safe, was well-tolerated, activated cGMP, induced natriuresis, reduced aldosterone, and decreased BP at or below the maximal tolerated dose. These exciting results support further investigations of ANP and its analogs as BP-lowering, natriuretic medications for hypertensive patients. As Volpe et al. noticed in their Commentary, “the clinical development of this innovative ANP-based, cGMP stimulating therapy, targeting a key mechanism in hypertension may provide a progress in ANP-based therapies and offer a new tool for the treatment of hypertension and its sequelae” (76). However, although these findings are encouraging, the mechanisms that underlie the potential effectiveness of ANP-based therapies require further studies.

## Methods

Supplemental Methods are available online with this article.

### Animals.

SS^NPPA–/–^ rats were generated at the Medical College of Wisconsin (12); the model was created on a Dahl SS rat background, which was used as a control (SS^WT^). Only male animals were used. Animals were kept on a AIN-76A based 0.4% NaCl, normal-salt diet (NS, Dyets Inc., 113755) unless otherwise indicated; food and water were provided ad libitum.

### Experimental protocols.

To induce SS hypertension, 8-week-old male SS^WT^ or SS^NPPA–/–^ rats were switched to a HS diet (4% NaCl; Dyets) for 21 days. In Protocol 1, infusion of ANP (100 ng/kg/day; continuous infusion at the rate of 10 mL/day through a venous catheter in saline) or vehicle (saline) was administered throughout the 21 days via an i.v. catheter, together with the HS diet. In Protocol 2, i.v. ANP or vehicle infusion was initiated at day 14 of the HS challenge. In order to install an i.v. catheter for ANP infusion and an arterial line for BP recording, polyvinyl catheters were implanted in the femoral artery and vein, tunneled s.c., and exteriorized at the back of the neck (77). BP was recorded throughout the protocol. Metabolic cage urine collections were done at days 0, 14, and 21 of the dietary challenge. In Protocol 2, to induce SS hypertension at the age of 8 weeks, male SS^WT^ and SS^NPPA–/–^ rats were switched to a HS diet for 21 days. An additional control group was kept on a NS diet. BP was recorded with telemetry (TA11 PA-C40, DSI) as done previously (78). MAP and HR were obtained daily continuously and are reported as 3-hour BP averages spanning from 9 a.m. until 12 p.m. All animals were euthanized on day 21 of the HS challenge by kidney flush via an abdominal aorta followed by a thoracotomy. Weights of nondehydrated organs were collected at the point of euthanasia.

### Metabolic cage balance studies, urinalysis, plasma electrolyte, and cGMP level measurements.

Urinary output was measured in metabolic cages for 24 hours. Urine and plasma electrolytes and creatinine were evaluated with a blood gas and electrolyte analyzer (ABL 800 Flex; Radiometer). Plasma was also sent for analysis to Marshfield Labs and Attoquant Diagnostic GmBH (Austria, RAAS metabolites panel). Urinary albumin, plasma ANP, and tissue cGMP were assessed with commercial kits (Ethos Biosciences, Nephrat ELISA, NR002; Ab108797, Abcam; no. 581021, Cayman Chemical).

### Glomerular filtration rate (GFR) measurement.

GFR was measured in unrestrained conscious rats using a FITC-labeled inulin (TdB Consultancy AB). The method was adapted from a protocol previously described (79). Animals were anesthetized briefly, and 2 μL/g of body weight of predialyzed 2% FITC-inulin was injected into a tail vein; inulin clearance from blood was quantified by FITC fluorescence intensity. GFR was then calculated using the previously described equations (80).

### Endpoint surgery and tissue isolations.

After the experimental protocol, rats were anesthetized, and the descending aorta was catheterized as done previously (81). Kidneys were flushed via the catheter with PBS until blanched; they were then excised and decapsulated. Tissues were immediately snap-frozen, fixed in 10% formalin, or used fresh in functional experiments.

### Histology, IHC, and tissue damage scoring.

Tissues (heart, kidney) were routinely fixed, embedded, and processed as done previously (82). Organs were randomized and coded before being submitted for blocking, sectioning, and staining, and researchers were blinded to groups when performing the analysis. Analysis of the tissue damage (protein casts, tubulointerstitial fibrosis) was assessed in FIJI (NIH; https://imagej.net) using the Color Deconvolution and Threshold tools used to calculate the percentage the total tissue area of a specific color (pertaining to protein casts [red/purple] or blue [fibrosis]). One whole organ section obtained in the middle of the organ stained with Masson trichrome was quantified per animal. IHC staining of the renal tissue for Megalin (antibodies were provided by F. Theilig, University of Kiel, Kiel, Germany; ref. 83) and αSMA (DAKO, M0851) was performed as described (77, 80) and analyzed in FIJI using color deconvolution and Intensity Profile plugins.

### Echocardiography.

Animals were weighed and anesthetized by isoflurane for transthoracic echocardiography. Echocardiography image acquisition and measurements will be made using a VIVID7 (GE Healthcare) ultrasound machine optimized for rodent imaging (84). Values from a 4 beat average were used as a data point for each animal.

### Patch-clamp electrophysiology.

Electrophysiological recordings were performed in the cell-attached patch-clamp mode of the voltage-clamp configuration. ENaC activity was measured in split-open collecting ducts isolated manually as described previously (21, 85). Recording solutions were as follows: extracellular (in mM) 150 NaCl, 1 MgCl_2_, 10 HEPES (pH 7.35); pipette solution (in mM): 140 LiCl, 2 MgCl_2_, and 10 HEPES (pH 7.35).

### Western blotting.

Cortical kidney pieces were lysed and subjected to SDS-PAGE, transferred onto a nitrocellulose membrane (Bio-Rad) for probing with antibodies, and subsequently visualized by enhanced chemiluminescence using standard protocols (ECL; Thermo Fisher Scientific). The following antibodies were used: BNP (Invitrogen, PA5-96084, 1:500), Megalin/LRP2 (Proteintech, 19700-1-AP, 1:1000), and goat anti–rabbit IgG secondary antibody HRP (Invitrogen, 31460). Total protein was assayed using Pierce stain.

### Statistics.

Differences among the experimental groups were measured using 1- or 2-way ANOVA, with Holm-Sidak post hoc, repeated measures ANOVA or 1- or 2-tailed Student’s *t* test, and log-rank test, as appropriate. Normality was tested in every experiment with a Kolmogorov-Smirnov test or equal variance (Levene’s homogeneity test). Data are presented as means ± SEM in full and analyzed in Excel and OriginPro software 2019b. In the box plot graphs, the box represents the mean ± SEM, and the mean value is marked by a horizontal line inside the box. The whiskers represent ± SD. *P* < 0.05 was considered significant. During histological analysis, investigators have been blinded to the sample group allocation during the analysis of the experimental outcome.

### Study approval.

Animal use and welfare adhered to the ARRIVE guidelines and *Guide for the Care and Use of Laboratory Animals* (National Academies Press, 2011) following a protocol reviewed and approved by the IACUC of the Medical College of Wisconsin.

## Author contributions

DVI and AS conceived the study and were responsible for the experimental design. DVI, VL, KW, GRB, DRS, ES, IP, AZ, MP, AJK, and AS took part in performing experiments, analyzing and interpreting data, and drafting and editing the manuscript. All authors have reviewed and approved the article.

## Supplementary Material

Supplemental data

Supplemental figures 1-3

Supplemental tables 1-4

## Version 1 04/05/2022

In-Press Preview

## Version 2 05/09/2022

Electronic publication
