# Supplementary material for: Effects of elevation of ANP and its deficiency on cardiorenal function
Source: JCI Insight. 2022 May 9;7(9):e148682. doi: 10.1172/jci.insight.148682 (PMC9090260; doi:10.1172/jci.insight.148682)
Supplement: Supplemental data [file jciinsight-7-148682-s123.pdf]

SUPPLEMENTARY FIGURE LEGENDS

SUPPLEMENTARY FIGURE S1. **(A)** Systolic and diastolic arterial pressures (matching MAP shown in **Figure 3B**) recorded via telemetry in SS^{WT} (N=6) vs $SS^{NPPA-/-}$ (N=7) rats throughout the experimental protocol. **(B)** Circadian rhythms of the blood pressure recorded via telemetry in SS^{WT} (N=6) vs $SS^{NPPA-/-}$ (N=7) rats on 3 days before the switch to a HS diet (left) and during the last 3 days of the protocol (right). Each point is an average of 4-hour period. Circadian rhythm analysis results (mesor, acrophase and amplitude) are shown in a table below the blood pressure curves; *** denotes p-value < 0.001. (**C**) Western blot analysis of BNP level (shown by an arrowhead, 15 kDa) in the plasma of the SS^{WT} and $SS^{NPPA-/-}$ animals on NS and HS diets; each lane is a sample from an independent experimental animals. Statistical analysis performed with two-way ANOVA with a Holm-Sidak post-hoc test.

SUPPLEMENTARY FIGURE S2. Representative FITC-inulin clearance curves used to quantify GFR (glomerular filtration rate). GFR was measured in 8 weeks old SS^{WT} and $SS^{NPPA-/-}$ rats on NS, and then in the same rats 20 days after switching to HS (N = 8, 7, 14, 12). Summary graph is shown on the right. Data was analyzed with 2-way ANOVA followed by a Holm-Sidak post hoc test; if found significant, p-values are shown on the graphs.

SUPPLEMENTARY FIGURE S3. Circadian rhythms of the heart rate recorded via telemetry in male SS^{WT} (N=6) vs $SS^{NPPA-/-}$ (N=7) rats on 3 days before the switch to a HS diet (left), and during the last 3 days of the protocol (right). Each point is an average of 6-hours period.

SUPPLEMENTARY TABLE LEGENDS

SUPPLEMENTARY TABLE S1. Urine and plasma parameters of the male SS^{WT} rats infused with ANP or vehicle at the end of the 21-day HS challenge. Body weight, daily urine flow, daily excretion of electrolytes (Na^+, K^+, and Cl^-) and plasma level of electrolytes (Na^+, K^+, and Cl^-) and creatinine of the male SS^{WT} rats administered vehicle, or ANP on days 14-21 and 0-21 of the HS challenge; N=17, 8, and 7 individual rats analyzed, respectively. Shown is data at the end of the 21-day period. Data was analyzed

with repeated measures ANOVA followed by a Holm-Sidak post hoc test. * denotes p-value <0.001, vehicle vs 0-21D infusion; **denotes p-value <0.001, vehicle vs 14-21D infusion; ‡ = p-value <0.001, 14-21D infusion vs 0-21D infusion.

SUPPLEMENTARY TABLE S2. Urine and plasma parameters of the male SS^{WT} and $SS^{NPPA-/-}$ rats; normal salt (NS) vs high salt (HS). Shown are body weight, daily excretion of K^+, Ca^{2+}, and creatinine, and plasma levels of total protein, globulin, albnumine/globuline ratio, alanine aminotransferase (ALT), alkaline phosphatase (Alk. Ptase), and cholesterol obtained from male SS^{WT} and $SS^{NPPA-/-}$ rats at 8 and 11 weeks of age (data obtained before and after the start of the HS challenge). Data was analyzed with 2-way ANOVA followed by a Holm-Sidak post hoc test. * denotes p-value <0.05, NS-SS^{WT} vs HS-SS^{WT}; ** = p-value <0.05, NS-$SS^{NPPA-/-}$ vs HS-$SS^{NPPA-/-}$; ‡ = p-value <0.05, HS-SS^{WT} vs HS-$SS^{NPPA-/-}$; ‡ ‡ = p-value <0.05, NS-SS^{WT} vs NS-$SS^{NPPA-/-}$.

SUPPLEMENTARY TABLE S3. Plasma parameters of the male SS^{WT} and $SS^{NPPA-/-}$ rats; normal salt (NS) vs high salt (HS). Plasma levels of Na^+, Cl^- and K^+, creatinine, BUN, obtained from male SS^{WT} and $SS^{NPPA-/-}$ rats at 11 weeks of age at the end of the 21-day long NS or HS protocol (N = 8, 11, 8 and 9, respectively, for creatinine and electrolytes, and N = 7 for each group for other parameters). Shown are also plasma levels of Angiotensin I (1-10), Angiotensin III (2-8), Angiotensin 1-7, Angiotensin 1-5, Angiotensin IV (3-8), and ACE activity (N = 5 tissue samples obtained from individual rats per group). Data was analyzed with 2-way ANOVA followed by a Holm-Sidak post hoc test. * denotes p-value <0.05, NS-SS^{WT} vs HS-SS^{WT}; ** p-value <0.05, NS-$SS^{NPPA-/-}$ vs HS-$SS^{NPPA-/-}$; ‡ p-value < 0.05, HS-SS^{WT} vs HS-$SS^{NPPA-/-}$; ‡ ‡ p-value < 0.05, NS-SS^{WT} vs NS-$SS^{NPPA-/-}$.

SUPPLEMENTARY TABLE S4. Echocardiography parameters of the male SS^{WT} and $SS^{NPPA-/-}$ rats; normal salt (NS) vs high salt (HS). Summary of various parameters from the echocardiography studies done in male SS^{WT} rats on normal satl (NS) and high salt (HS), and $SS^{NPPA-/-}$ rats on NS and HS (N = 10, 8, 11, 14 respectively). Data was analyzed with repeated measures ANOVA. LV – left ventricle; RV –

right ventricle. * p-value <0.05, NS-SS^{WT} vs HS-SS^{WT}; ** p-value <0.05, NS-SS$^{NPPA-/-}$ vs HS-SS$^{NPPA-/-}$; ‡p-value < 0.05, HS-SS^{WT} vs HS-SS$^{NPPA-/-}$; ‡ ‡ p-value < 0.05, NS-SS^{WT} vs NS-SS$^{NPPA-/-}$.

EXPANDED METHODS

Animals

Animal use and welfare adhered to the ARRIVE guidelines and NIH Guide for the Care and Use of Laboratory Animals following a protocol reviewed and approved by the IACUC of the Medical College of Wisconsin. *Nppa* knockout rats (SS$^{NPPA-/-}$) were generated at the Medical College of Wisconsin using zinc finger nuclease mutagenesis;[18] the model was created on a Dahl SS rat background, which was used as a control (SS^{WT}). Animals were kept on an AIN-76A based 0.4% NaCl normal salt diet (NS, Dyets Inc, #113755) unless otherwise indicated; food and water were provided *ad libitum*. Both male and female rats were used; sex and age of animals defined in respective protocols.

Experimental Protocols. Animals were kept on 0.4% NaCl diet (see above), unless SS hypertension was induced. In order to induce hypertension, 8-week-old male or female rats were switched to a high salt diet (HS, 4% NaCl; Dyets) for 21 days.

In Protocol 1, infusion of ANP (100 ng/kg/day; continuous infusion at the rate of 6.9 µl/min through a venous catheter in saline as a vehicle) or vehicle was administered throughout the 21 days via an *i.v.* catheter, together with the HS diet. In order to install *i.v.* catheter for ANP infusion, and an arterial line for blood pressure recording, polyvinyl catheters were implanted in the femoral artery and vein, tunneled subcutaneously, and exteriorized at the back of the neck.[27] Blood pressure was recorded throughout the protocol. Metabolic cage urine collections were done at day 0, 14, and 21 of the dietary challenge; *i.v.* ANP infusion was initiated at day 1 or day 14 of the HS challenge.

Protocol 2. To induce salt-sensitive hypertension at the age of 8 weeks, male and female SS^{WT} and SS$^{NPPA-/-}$ rats were switched to a HS diet for 21 days. An additional control group was kept on a NS diet. Blood pressure was recorded with telemetry (#TA11 PA-C40, DSI, MN) as done previously.[28] Mean

arterial pressure (MAP) and heart rate (HR) were obtained daily 24 hrs/d, and are reported as an 3 hrs average from 9 AM till noon readings. In Protocol 2, *i.v.* ANP or vehicle infusion were initiated at day 14 of the HS challenge. For analyzing circadian rhythms in blood pressure, MAP from the last three days of the NS and HS diets was averaged hourly over 24-hrs, starting from the beginning of the light cycle (6 am), and cosinor analysis was performed using Microsoft Excel. Once mesor, amplitude, and acrophase had been calculated, the values for each of the three days of NS and HS were averaged for both SSWT and SSNPPA-/- rats and were compared using two-way ANOVA.

Metabolic Cage Balance Studies, Urinalysis, Plasma Electrolyte, and cGMP Level Measurements

For the continuous urine collection rats were put in metabolic cages, and urinary output was measured for 24 hours following a 1-day long acclimation period. Urine and plasma electrolytes and creatinine were evaluated with a blood gas and electrolyte analyzer (ABL system 800 Flex; Radiometer, Copenhagen, Denmark). This radiometer records electrolyte levels based on a potentiometric measurement system; electrolyte levels were normalized to daily urine flow and animal weight. In addition, plasma was sent for analysis to Marshfield veterinary labs (WI, USA: plasma albumin, globulin, BUN, ALT, AlkP, cholesterol, total protein, creatinine) and Attoquant Diagnostic GmBH (Austria, RAAS metabolites panel). Urinary albumin level was assessed with a commercially available kit according to the manufacturer’s instructions (Ethos Biosciences, Nephrat ELISA #NR002). cGMP level was assessed in the renal tissue using a commercially available kit (No. 581021, Cayman Chemical, USA). Rat ANP ELISA kit (Ab108797, Abcam, USA) was employed for plasma ANP level measurements according to vendor’s recommendations.

Glomerular Filtration Rate (GFR) Measurement

The glomerular filtration rate (GFR) was measured in unrestrained conscious rats using a high-throughput method featuring detection of fluorescent FITC-labeled inulin (TdB Consultancy AB, Uppsala, Sweden) clearance from blood. The method was adapted from a protocol described.[29] Body weight of rats was recorded before the experiment. Animals were anesthetized briefly, and 2 µl/g of body weight of pre-

dialyzed 2% FITC-inulin was injected into a tail vein. Immediately after the injection, anesthesia was discontinued. 10 µl of blood was collected at 3, 5, 8, 16, 25, 40, 60 and 80 min after injection, and inulin clearance was quantified by FITC fluorescence intensity using a NanoDrop 3300 Fluorospectrometer (Thermo Fisher Scientific, Wilmington, DE, USA). GFR was then calculated from the observed decrease in FITC fluorescence using a two-compartment model, and the GFR curve was approximated with a bi-exponential decay function using Origin 7.0 (OriginLab, Northhampton, MA) software GFR was then calculated using the previously described equations.[30]

Endpoint Surgery and Tissue Isolations

At the completion of the experimental protocol, rats were anesthetized, and descending aorta was catheterized as done previously.[31] Kidneys were flushed via the catheter with PBS (3 ml/min/kidney) until blanched, then excised, and the capsule was removed. Tissues were then immediately collected, and either snap-frozen in liquid nitrogen, fixed in 10% formalin, or used fresh in functional experiments. Tissue and body weights were recorded.

Histology, Immunohistochemistry and Tissue Damage Scoring

Tissues (heart, kidney) were routinely fixed, embedded and processed as done previously.[32] Organs were randomized and coded before being submitted for blocking, sectioning, and staining. Slides were stained with the routine Masson's trichrome stain, and images were obtained on Nikon E-400 or Nikon Ti-2 microscopes. Analysis of the tissue damage (glomerular damage score, protein cast formation, interstitial and perivascular fibrosis) was assessed in FIJI. Immunohistochemical staining of the renal tissue for Megalin (abs were provided by Dr. Theilig, University of Kiel, Germany[33]) and αSMA (DAKO M0851) was performed as described[27,30] and analyzed in FIJI using color deconvolution plugins.

Echocardiography

Animals were weighed and anesthetized by isoflurane for transthoracic echocardiography. Echocardiography image acquisition and measurements will be made using a VIVID7 (GE) ultrasound machine optimized for rodent imaging.[34] Values from a 4 beat average will be used as a data point for

each animal. The images of the left ventricle in the short axis, mid-papillary region will be analyzed to obtain the following measurements: dorsal and ventral wall diastolic and systolic wall thickness, diastolic and systolic chamber diameter, heart rate, percent fractional shortening, estimated ejection fraction, and estimated stroke volume. Short axis images of the right ventricle will also be analyzed to study changes in chamber diameter in systole and diastole.

Patch-Clamp Electrophysiology

Electrophysiological recordings were performed in the cell-attached patch-clamp mode of the voltage-clamp configuration. ENaC activity was measured in split-open collecting ducts (CD) isolated manually as described previously.[21,35] Recording solutions were as follows: extracellular (in mM) 150 NaCl, 1 $MgCl_2$, 10 HEPES (pH 7.35); pipette solution (in mM): 140 LiCl, 2 $MgCl_2$, and 10 HEPES (pH 7.35). Resistances of patch pipettes ranged from 7 to 12 MΩ. After a high resistance seal was obtained, the cell-attached recordings were performed immediately and analyzed as described previously.[21,35]

Statistical Analysis

Differences among the experimental groups were measured using 1 or 2-way ANOVA, with Holm-Sidak post-hoc, repeated measures ANOVA or Student's t-test, as appropriate. Normality was tested in every experiment with a Kolmogorov–Smirnov test or equal variance (Levene's homogeneity test). Data are presented as means ± SEM in full and analyzed in Excel and OriginPro software 2019b. In the box plot graphs, the box represents SE, and the mean value is marked by a horizontal line inside the box. The whiskers are ± SD; p-value of <0.05 was considered significant.
