# Supplementary material for: Effects of elevation of ANP and its deficiency on cardiorenal function
Source: JCI Insight. 2022 May 9;7(9):e148682. doi: 10.1172/jci.insight.148682 (PMC9090260; doi:10.1172/jci.insight.148682)
Supplement: Supplemental figures 1-3 [file jciinsight-7-148682-s124.pptx]

## Slide 1
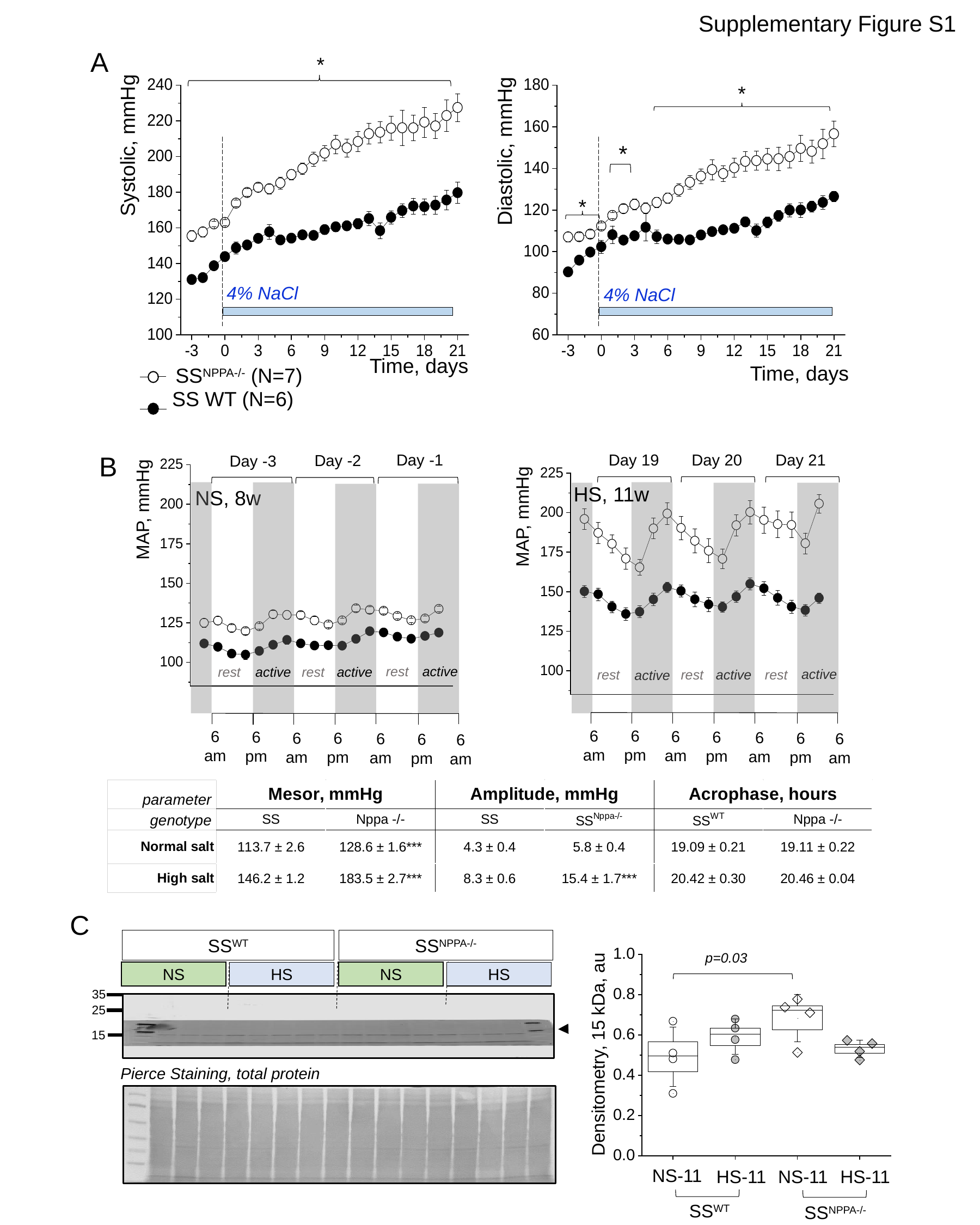

Supplementary Figure S1
A
*
*
*
Systolic, mmHg
Diastolic, mmHg
*
4% NaCl
4% NaCl
Time, days
Time, days
SSNPPA-/- (N=7)
SS WT (N=6)
B
Day -1
Day 21
Day 19
Day 20
Day -2
Day -3
HS, 11w
NS, 8w
MAP, mmHg
MAP, mmHg
active
rest
active
rest
active
rest
active
rest
rest
active
rest
active
6 am
6 pm
6 am
6 am
6 pm
6 pm
6 am
6 am
6 pm
6 pm
6 am
6 am
6 pm
6 am
C
SSWT
SSNPPA-/-
p=0.03
NS
NS
HS
HS
35
25
15
Pierce Staining, total protein
Densitometry, 15 kDa, au
NS-11
HS-11
HS-11
NS-11
SSWT
SSNPPA-/-

## Slide 2
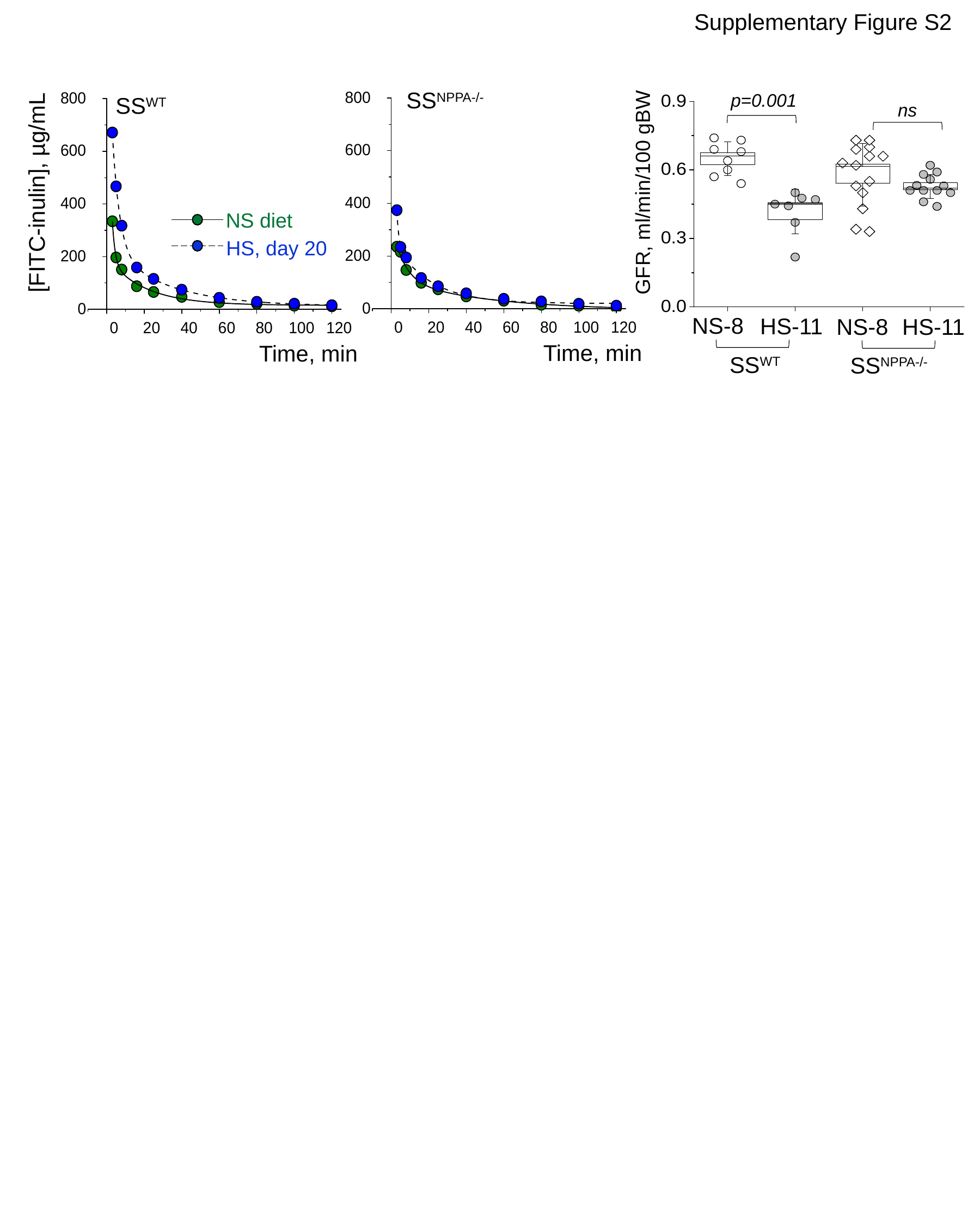

Supplementary Figure S2
SSNPPA-/-
SSWT
[FITC-inulin], µg/mL
NS diet
HS, day 20
Time, min
Time, min
p=0.001
ns
GFR, ml/min/100 gBW
NS-8
HS-11
HS-11
NS-8
SSWT
SSNPPA-/-

## Slide 3
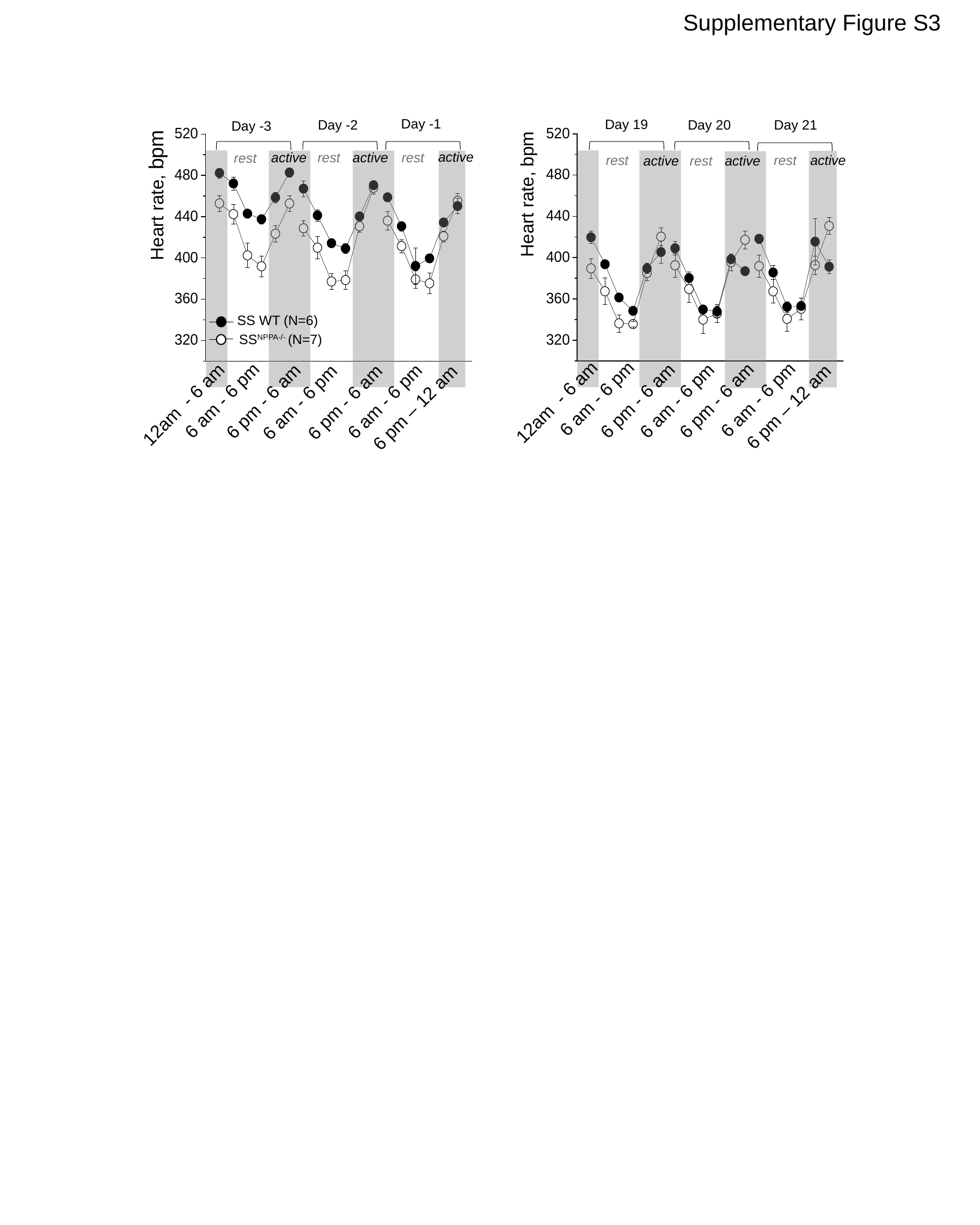

Supplementary Figure S3
Day -1
Day 19
Day -2
Day 20
Day 21
Day -3
Heart rate, bpm
Heart rate, bpm
SS WT (N=6)
SSNPPA-/- (N=7)
6 am - 6 pm
6 am - 6 pm
6 pm – 12 am
12am - 6 am
6 am - 6 pm
6 am - 6 pm
6 pm - 6 am
6 pm - 6 am
6 pm – 12 am
6 pm - 6 am
6 am - 6 pm
6 am - 6 pm
6 pm - 6 am
12am - 6 am
active
rest
active
rest
active
rest
active
rest
rest
active
rest
active
