# Supplementary material for: Effects of elevation of ANP and its deficiency on cardiorenal function
Source: JCI Insight. 2022 May 9;7(9):e148682. doi: 10.1172/jci.insight.148682 (PMC9090260; doi:10.1172/jci.insight.148682)
Supplement: Supplemental tables 1-4 [file jciinsight-7-148682-s125.pptx]

## Slide 1
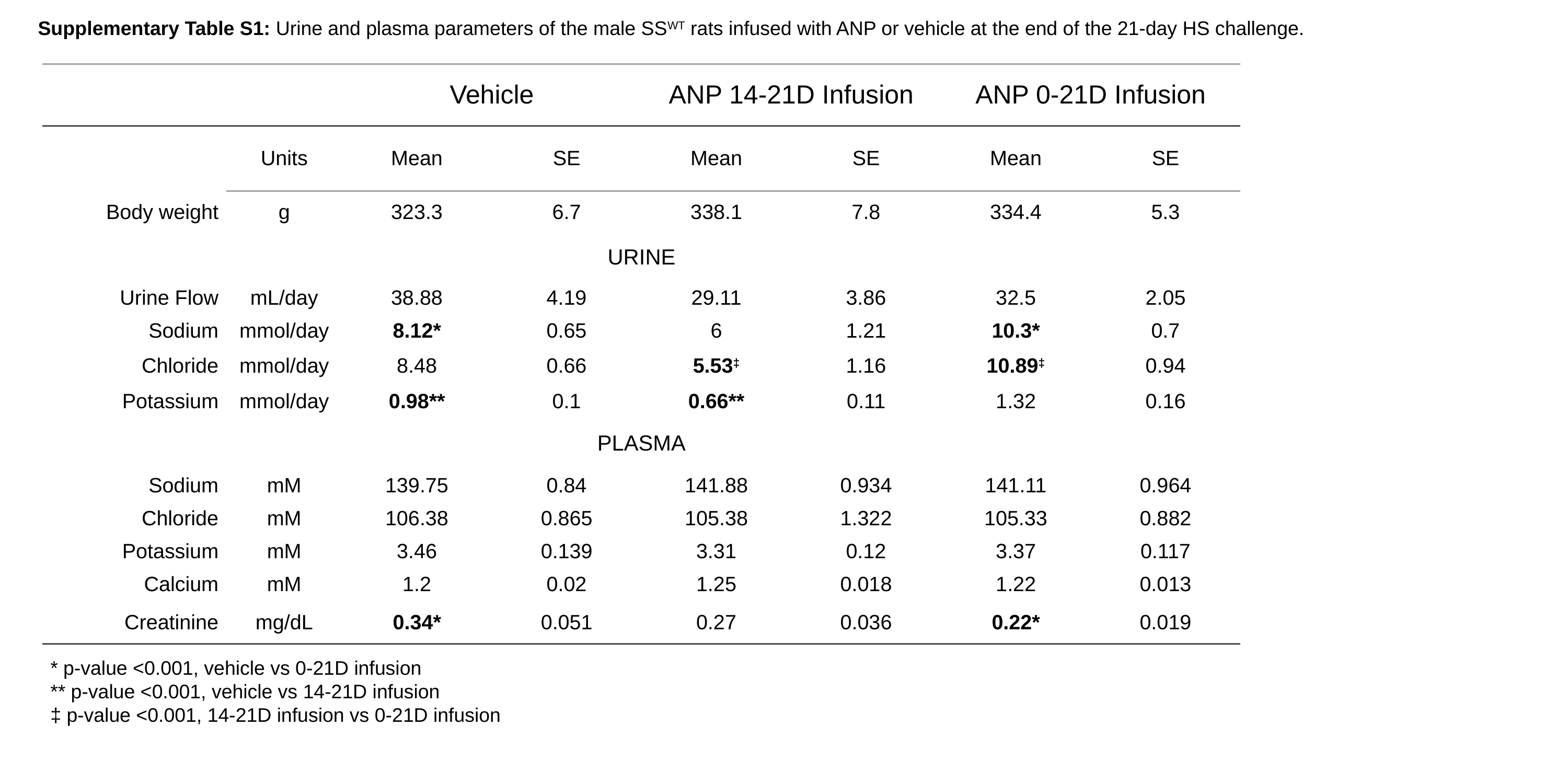

Supplementary Table S1: Urine and plasma parameters of the male SSWT rats infused with ANP or vehicle at the end of the 21-day HS challenge.
| | | Vehicle | | ANP 14-21D Infusion | | ANP 0-21D Infusion | |
| --- | --- | --- | --- | --- | --- | --- | --- |
| | Units | Mean | SE | Mean | SE | Mean | SE |
| Body weight | g | 323.3 | 6.7 | 338.1 | 7.8 | 334.4 | 5.3 |
| URINE | | | | | | | |
| Urine Flow | mL/day | 38.88 | 4.19 | 29.11 | 3.86 | 32.5 | 2.05 |
| Sodium | mmol/day | 8.12\* | 0.65 | 6 | 1.21 | 10.3\* | 0.7 |
| Chloride | mmol/day | 8.48 | 0.66 | 5.53‡ | 1.16 | 10.89‡ | 0.94 |
| Potassium | mmol/day | 0.98\*\* | 0.1 | 0.66\*\* | 0.11 | 1.32 | 0.16 |
| PLASMA | | | | | | | |
| Sodium | mM | 139.75 | 0.84 | 141.88 | 0.934 | 141.11 | 0.964 |
| Chloride | mM | 106.38 | 0.865 | 105.38 | 1.322 | 105.33 | 0.882 |
| Potassium | mM | 3.46 | 0.139 | 3.31 | 0.12 | 3.37 | 0.117 |
| Calcium | mM | 1.2 | 0.02 | 1.25 | 0.018 | 1.22 | 0.013 |
| Creatinine | mg/dL | 0.34\* | 0.051 | 0.27 | 0.036 | 0.22\* | 0.019 |
* p-value <0.001, vehicle vs 0-21D infusion
** p-value <0.001, vehicle vs 14-21D infusion
‡ p-value <0.001, 14-21D infusion vs 0-21D infusion

## Slide 2
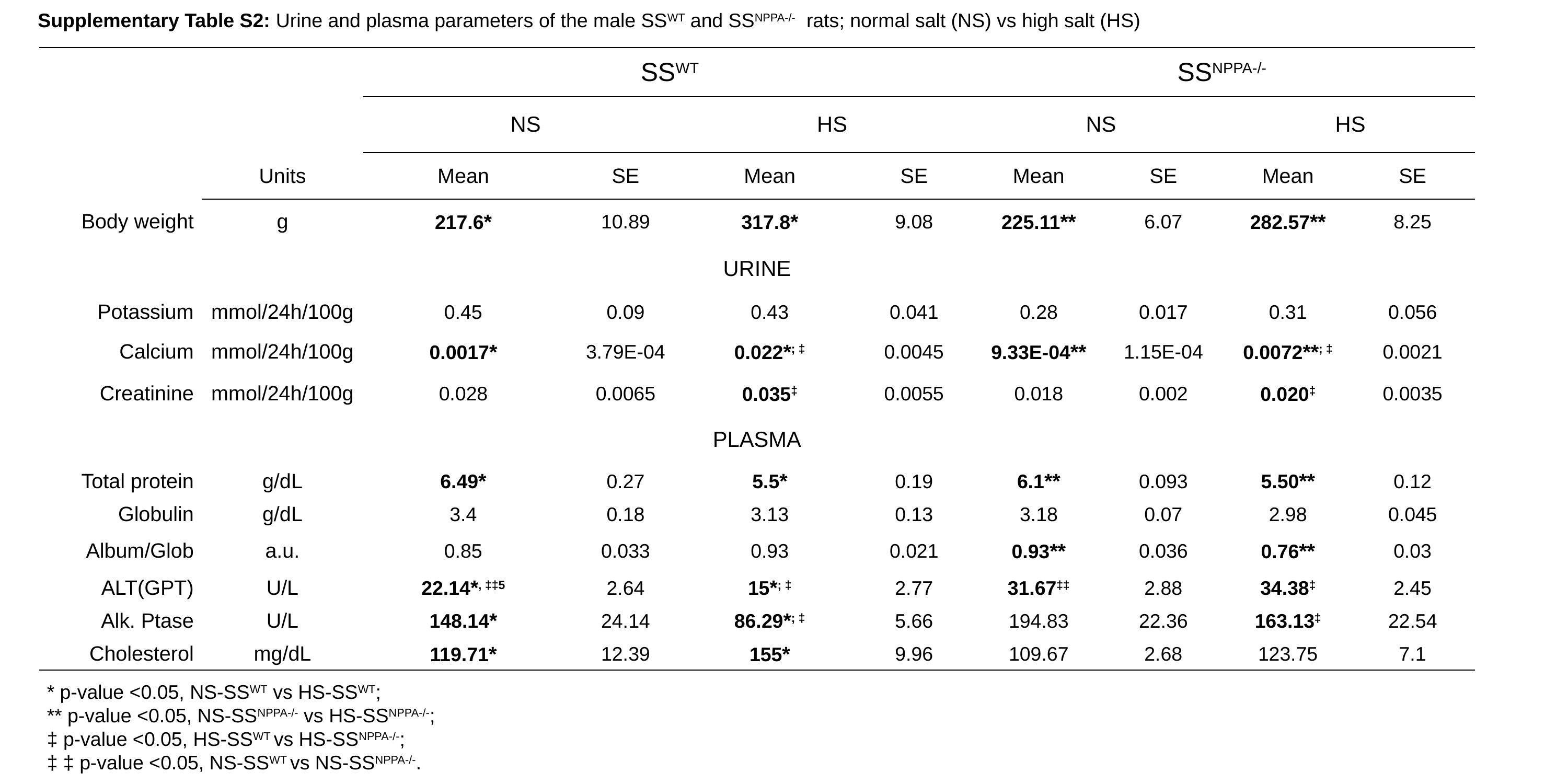

Supplementary Table S2: Urine and plasma parameters of the male SSWT and SSNPPA-/- rats; normal salt (NS) vs high salt (HS)
| | | SSWT | | | | SSNPPA-/- | | | |
| --- | --- | --- | --- | --- | --- | --- | --- | --- | --- |
| | | NS | | HS | | NS | | HS | |
| | Units | Mean | SE | Mean | SE | Mean | SE | Mean | SE |
| Body weight | g | 217.6\* | 10.89 | 317.8\* | 9.08 | 225.11\*\* | 6.07 | 282.57\*\* | 8.25 |
| URINE | | | | | | | | | |
| Potassium | mmol/24h/100g | 0.45 | 0.09 | 0.43 | 0.041 | 0.28 | 0.017 | 0.31 | 0.056 |
| Calcium | mmol/24h/100g | 0.0017\* | 3.79E-04 | 0.022\*; ‡ | 0.0045 | 9.33E-04\*\* | 1.15E-04 | 0.0072\*\*; ‡ | 0.0021 |
| Creatinine | mmol/24h/100g | 0.028 | 0.0065 | 0.035‡ | 0.0055 | 0.018 | 0.002 | 0.020‡ | 0.0035 |
| PLASMA | | | | | | | | | |
| Total protein | g/dL | 6.49\* | 0.27 | 5.5\* | 0.19 | 6.1\*\* | 0.093 | 5.50\*\* | 0.12 |
| Globulin | g/dL | 3.4 | 0.18 | 3.13 | 0.13 | 3.18 | 0.07 | 2.98 | 0.045 |
| Album/Glob | a.u. | 0.85 | 0.033 | 0.93 | 0.021 | 0.93\*\* | 0.036 | 0.76\*\* | 0.03 |
| ALT(GPT) | U/L | 22.14\*, ‡‡5 | 2.64 | 15\*; ‡ | 2.77 | 31.67‡‡ | 2.88 | 34.38‡ | 2.45 |
| Alk. Ptase | U/L | 148.14\* | 24.14 | 86.29\*; ‡ | 5.66 | 194.83 | 22.36 | 163.13‡ | 22.54 |
| Cholesterol | mg/dL | 119.71\* | 12.39 | 155\* | 9.96 | 109.67 | 2.68 | 123.75 | 7.1 |
* p-value <0.05, NS-SSWT vs HS-SSWT;
** p-value <0.05, NS-SSNPPA-/- vs HS-SSNPPA-/-;
‡ p-value <0.05, HS-SSWT vs HS-SSNPPA-/-;
‡ ‡ p-value <0.05, NS-SSWT vs NS-SSNPPA-/-.

## Slide 3
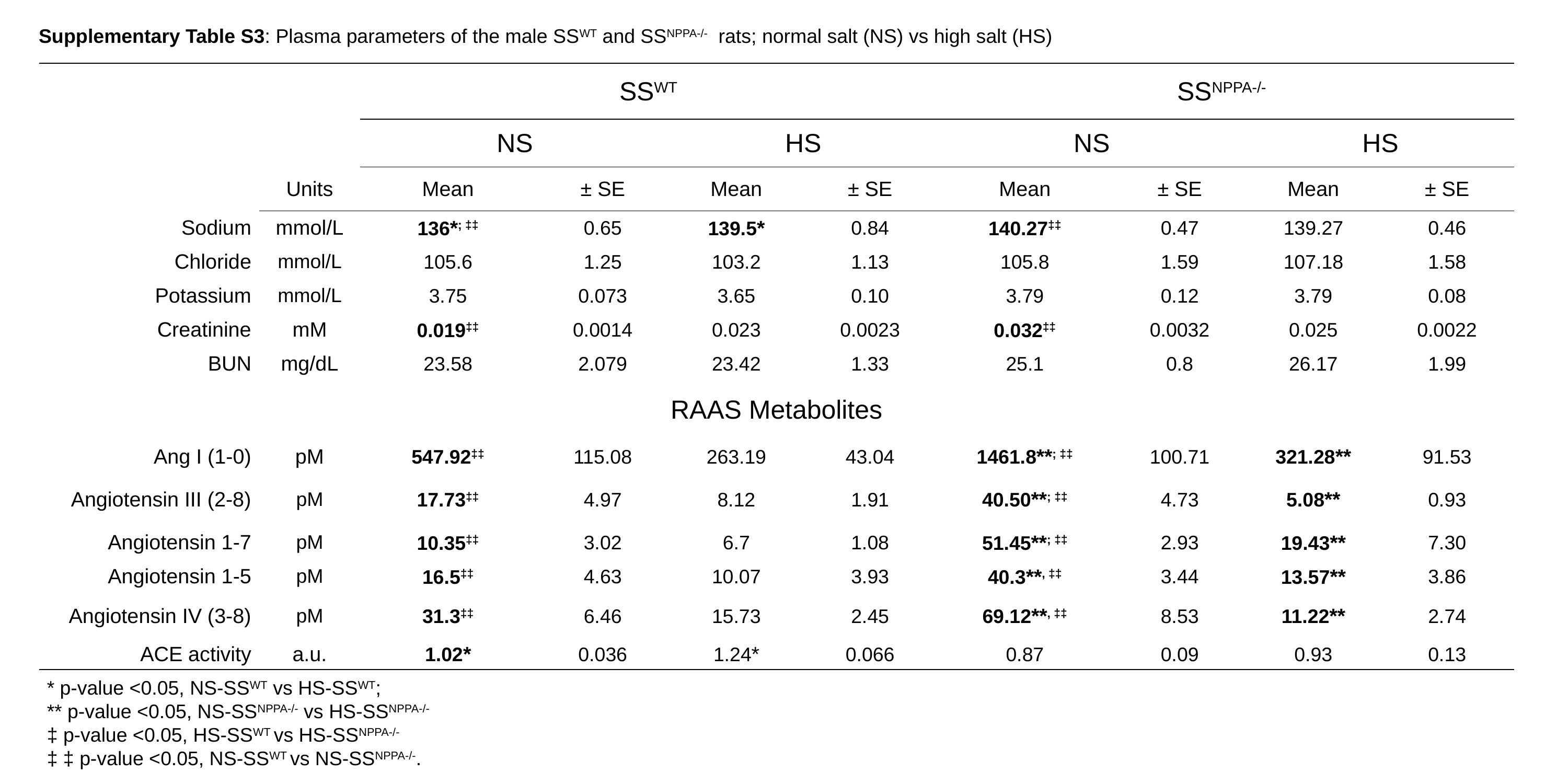

Supplementary Table S3: Plasma parameters of the male SSWT and SSNPPA-/- rats; normal salt (NS) vs high salt (HS)
| | | SSWT | | | | SSNPPA-/- | | | |
| --- | --- | --- | --- | --- | --- | --- | --- | --- | --- |
| | | NS | | HS | | NS | | HS | |
| | Units | Mean | ± SE | Mean | ± SE | Mean | ± SE | Mean | ± SE |
| Sodium | mmol/L | 136\*; ‡‡ | 0.65 | 139.5\* | 0.84 | 140.27‡‡ | 0.47 | 139.27 | 0.46 |
| Chloride | mmol/L | 105.6 | 1.25 | 103.2 | 1.13 | 105.8 | 1.59 | 107.18 | 1.58 |
| Potassium | mmol/L | 3.75 | 0.073 | 3.65 | 0.10 | 3.79 | 0.12 | 3.79 | 0.08 |
| Creatinine | mM | 0.019‡‡ | 0.0014 | 0.023 | 0.0023 | 0.032‡‡ | 0.0032 | 0.025 | 0.0022 |
| BUN | mg/dL | 23.58 | 2.079 | 23.42 | 1.33 | 25.1 | 0.8 | 26.17 | 1.99 |
| RAAS Metabolites | | | | | | | | | |
| Ang I (1-0) | pM | 547.92‡‡ | 115.08 | 263.19 | 43.04 | 1461.8\*\*; ‡‡ | 100.71 | 321.28\*\* | 91.53 |
| Angiotensin III (2-8) | pM | 17.73‡‡ | 4.97 | 8.12 | 1.91 | 40.50\*\*; ‡‡ | 4.73 | 5.08\*\* | 0.93 |
| Angiotensin 1-7 | pM | 10.35‡‡ | 3.02 | 6.7 | 1.08 | 51.45\*\*; ‡‡ | 2.93 | 19.43\*\* | 7.30 |
| Angiotensin 1-5 | pM | 16.5‡‡ | 4.63 | 10.07 | 3.93 | 40.3\*\*, ‡‡ | 3.44 | 13.57\*\* | 3.86 |
| Angiotensin IV (3-8) | pM | 31.3‡‡ | 6.46 | 15.73 | 2.45 | 69.12\*\*, ‡‡ | 8.53 | 11.22\*\* | 2.74 |
| ACE activity | a.u. | 1.02\* | 0.036 | 1.24\* | 0.066 | 0.87 | 0.09 | 0.93 | 0.13 |
* p-value <0.05, NS-SSWT vs HS-SSWT;
** p-value <0.05, NS-SSNPPA-/- vs HS-SSNPPA-/-
‡ p-value <0.05, HS-SSWT vs HS-SSNPPA-/-
‡ ‡ p-value <0.05, NS-SSWT vs NS-SSNPPA-/-.

## Slide 4
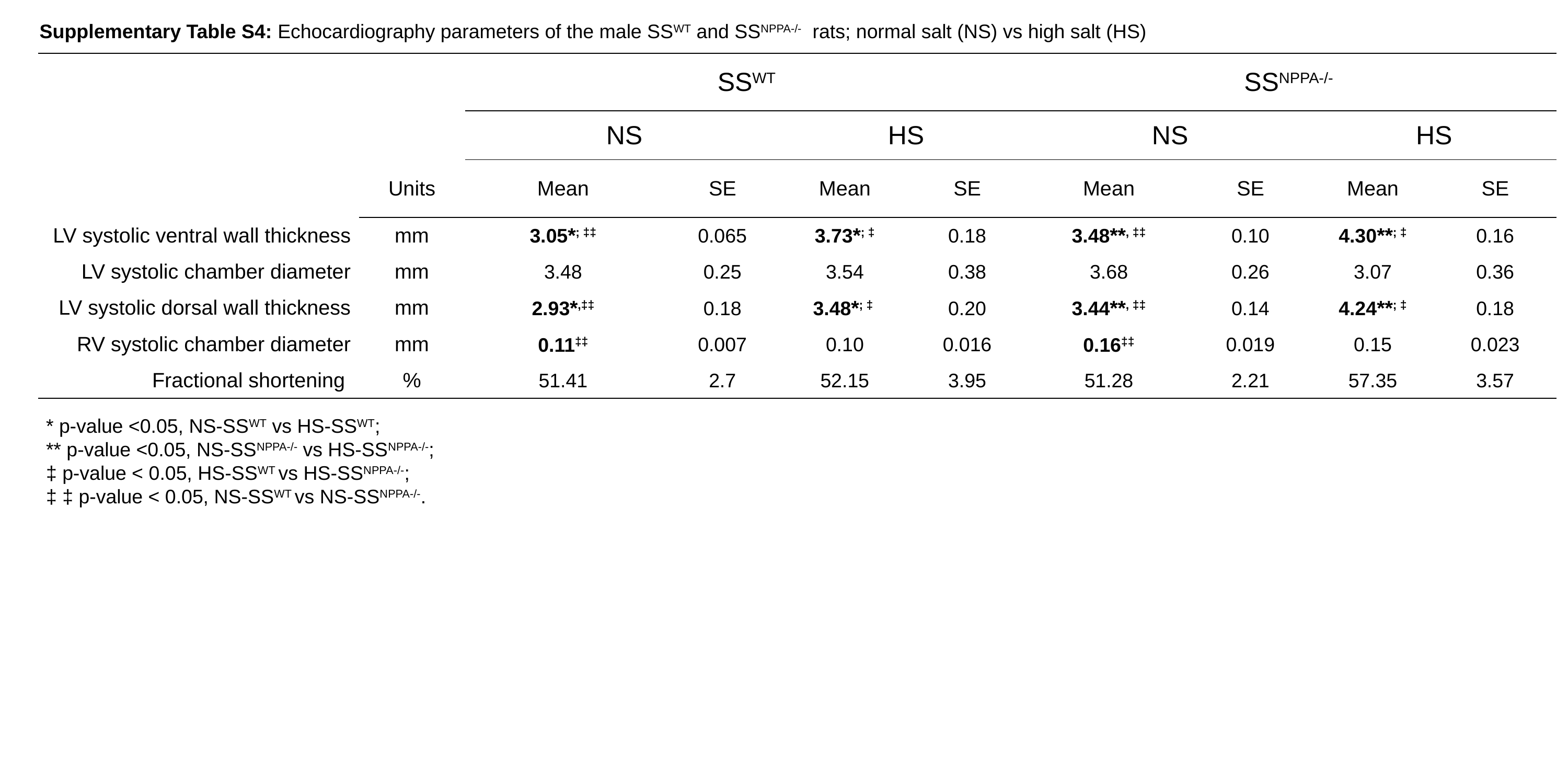

Supplementary Table S4: Echocardiography parameters of the male SSWT and SSNPPA-/- rats; normal salt (NS) vs high salt (HS)
| | | SSWT | | | | SSNPPA-/- | | | |
| --- | --- | --- | --- | --- | --- | --- | --- | --- | --- |
| | | NS | | HS | | NS | | HS | |
| | Units | Mean | SE | Mean | SE | Mean | SE | Mean | SE |
| LV systolic ventral wall thickness | mm | 3.05\*; ‡‡ | 0.065 | 3.73\*; ‡ | 0.18 | 3.48\*\*, ‡‡ | 0.10 | 4.30\*\*; ‡ | 0.16 |
| LV systolic chamber diameter | mm | 3.48 | 0.25 | 3.54 | 0.38 | 3.68 | 0.26 | 3.07 | 0.36 |
| LV systolic dorsal wall thickness | mm | 2.93\*,‡‡ | 0.18 | 3.48\*; ‡ | 0.20 | 3.44\*\*, ‡‡ | 0.14 | 4.24\*\*; ‡ | 0.18 |
| RV systolic chamber diameter | mm | 0.11‡‡ | 0.007 | 0.10 | 0.016 | 0.16‡‡ | 0.019 | 0.15 | 0.023 |
| Fractional shortening | % | 51.41 | 2.7 | 52.15 | 3.95 | 51.28 | 2.21 | 57.35 | 3.57 |
* p-value <0.05, NS-SSWT vs HS-SSWT;
** p-value <0.05, NS-SSNPPA-/- vs HS-SSNPPA-/-;
‡ p-value < 0.05, HS-SSWT vs HS-SSNPPA-/-;
‡ ‡ p-value < 0.05, NS-SSWT vs NS-SSNPPA-/-.
